# Trump’s Trade Policy, BREXIT, Corona Dynamics, EU Crisis and Declining Multilateralism

**DOI:** 10.1007/s10368-020-00479-x

**Published:** 2020-08-01

**Authors:** Paul J. J. Welfens

**Affiliations:** 1 European Institute for International Economic Relations, University of Wuppertal, Wuppertal, Germany; 2 AICGS/Johns Hopkins University, Washington DC, USA

**Keywords:** Trade policy, Brexit, Corona, EU, Multilateralism, F13, F00, I10, E61, E62

## Abstract

Since 1991 there has been a reinforcement of the World Market Economy, not least since China and the, then new Russian Federation have joined the World Trade Organization and because of EU Eastern enlargement and ASEAN integration deepening, while the Transatlantic Trade and Investment Partnership (TTIP) and Trans-Pacific Partnership (TPP) negotiations seemed to indicate stronger regional integration dynamics. With the Trump Administration, the situation has changed dramatically as President Trump is supportive of neither multilateralism in general nor of the EU, which is weakened through BREXIT, in particular. Trump’s focus on the US merchandise trade balance deficit is ill-placed and import tariffs imposed on China seem to be excessive as the optimum tariff rate is miscalculated on the basis of the traditional formula – while a new adequate formula would include the role of sectoral US outward FDI stocks. Asia, the EU and the US could define fighting the Corona World Recession as a global public good, but the United States is weakened in the corona pandemic crisis; the EU is facing serious problems in avoiding a Euro Crisis 2 problem and the €750 billion EU loan package could undermine the Eurozone’s stability while being inadequate to minimize the risk of a Euro Crisis 2. At the same time, the prospects for EU cooperation are declining due to political disappointment concerning the national corona pandemic policy in some member countries. An effective anti-corona pandemic policy would mean to organize a consistent EU-ASEAN cooperation or a G20 cooperation with a later extension to UN Organizations, including the IMF, the World Bank and the WHO. Post-corona, global governance could change strongly because of the long-term political scarring effects of the pandemic shock which could undermine EU and Western stability. Networked international leadership in support of multilateralism is an innovative – but difficult - option for EU-ASEAN-Mercosur**.**

## Introduction

The world economy has greatly changed since the end of the Cold War – the rise of China since the 1980s, the expansion of the digital economy worldwide since the 1990s, and a rising wave of populism and the declining political cohesion of the Western world since 2016 are visible as new traits of the new global order, while systemic competition among Western countries and the role of climate policy at a global scale has been reinforced; at the same time, multilateralism and the role of International Organizations, respectively, seem to have weakened. While in the EU significant political focus has been placed on the rise of China, the perception of the parallel rise of the ASEAN group, has been rather modest even though at the end of 2015 the ASEAN countries have embraced the start of an ASEAN single market and ASEAN will naturally also become a more important partner for the EU if US-Sino politico-economic conflicts should increase over time. While the US and China signed a first trade agreement in January 2020, its implementation is rather doubtful since the coronavirus shock has brought about a truly global recession – with output falling more than in the Transatlantic Banking Crisis of 2008. The Corona World Recession takes place in a strange setting, namely with China being a key part of an international economic crisis for the first time, while the traditional leadership which was present in every international crisis since 1945 is not visible at all: President Donald Trump, as a populist leader, is neither willing nor able to exert US leadership and this in turn is a new political shock to the Western world as will be argued subsequently.

The whole of the Western world is currently weakened, as not only is the US showing no leadership, but 2020 is also the year in which the government of Prime Minister Boris Johnson in the United Kingdom intends to proudly implement a BREXIT project which is inflicting massive economic costs on both the UK and the EU27; the UK’s performance – under the Johnson government - in fighting the Corona Pandemic is remarkably weak (the situation in the US is not any better, as more than 110,000 COVID-19 related death cases reveals a weak position for the world’s superpower in mid-2020). In Brussels, the Juncker Commission did not understand the challenge of the British EU referendum of 2016 and it is unclear whether the von der Leyen Commission will be able to keep the EU27 together in the face of a difficult COVID-19 public health and economic shock in 2020 – EU policies suggested by mid-2020 were indeed not likely to be adequate to avoid a new Euro Crisis 2 which will become acute once Italy should lose its investor grade ratings. Such downgrading in OECD countries is likely in a transatlantic environment in which the Trump Administration’s decisions set the course for a raising of the debt-GDP ratio from 100% in 2019 to about 160% in 2025.

A global recession means that many multinational companies will face much lower domestic sales. This in turn brings negative international investment and sales spillovers, if one is to follow recent analysis on the topic of international MNC supply-side spillovers (Cravino and Levchenko 2017). There are additional MNC spillovers in the Corona World Recession as intermediate product trade has been hampered by the introduction of new border controls and explicit new protectionism – including export barriers - in the first half of 2020: according to the University of St. Gallen database on protectionism, 75 countries have introduced barriers to the export of medical supplies, pharmaceuticals and epidemic-related equipment in early 2020 (FT, 2020). The Trump Administration has tried to promote exports in 2017–2020, partly through a special tax reform, and by threatening to impose import tariffs it has tried to promote inward FDI flows from OECD countries in a rather artificial way. By contrast, the US has tried to discourage Chinese FDI inflows and to limit, through new export barriers, international technology flows from the US to China. The US-Sino trade conflict involves the world’s leading two economies and therefore is bound to have negative spillovers affecting the rest of the world which has not yet adjusted to the new global regime of the global interdependency of big economies. ASEAN countries face enhanced competition for Western countries’ and China’s economic influence in the medium term; once the Corona World Recession has been overcome. The global economic picture is rather complex since 2016 and not enough analysis is available thus far to cover the new challenges. The following reflections will contribute several new thoughts, both related to theoretical and institutional analysis – in the end, one gets a post-corona picture of the world economy which suggests that more political instability, less regional integration and a weakening of Western democracies could be a key impact of the dynamics since about 2005; the extent to which fading US leadership could be replaced by a new networked approach can be discussed only in a first approach where EU-ASEAN cooperation could play a key role under certain circumstances. A crucial theoretical contribution will highlight the optimum tariff literature where one can show that the traditional result is not valid if there is outward cumulated foreign direct investment – and, in this perspective, the Trump Administration’s import tax rates imposed on China are all likely to be too high (assuming that this administration uses the traditional optimum tariff literature). That the Western world’s leading economy has no trade experts in government which would have noticed this analytical problem tells its own story.

ASEAN countries could benefit from rising US, British, EU and Chinese foreign direct investment inflows. This would encourage broader human capital formation and R&D capital accumulation plus the dynamics of the ASEAN single market, the perspectives for closer EU-ASEAN economic relations and political cooperation could become more important. Part of China’s investment in the land-based and the maritime new silk roads could also stimulate more EU-ASEAN trade. Moreover, with digital progress rising in the corona crisis, and expected to remain high worldwide in a post-corona setting, the EU’s interest in ASEAN digital economic capabilities – which are particularly strong, for example, in Singapore, Malaysia, parts of Indonesia and Thailand – could be reinforced.

The corona epidemic has changed the picture of international economic relations; the US-Sino trade conflict has intensified even more and ASEAN countries will have to consider what BREXIT – the UK’s departure from the European Union on January 31, 2020 – means for both the relations with the EU and with the UK. The May government had already pushed for new free trade treaties with ASEAN countries, but these refused to negotiate and wanted to know not only the final date of BREXIT implementation but also how the EU27-UK treaty regarding the future relationship would look. While the new Johnson government has announced that the negotiations with the EU will have to be finalized by the end of 2020 – even though the corona shock has now made the picture more complex – the European Union might not be inclined to soften its position since, in mid-2020, the re-election of Donald Trump looks not to be very likely. ASEAN countries will have to wait at least until 2021 to see what type of UK-EU27 trade treaty will be on the table.

The November 2020 US presidential election is a crucial element in the EU-UK negotiations. Without the populist US President Trump, the British negotiation position vis-à-vis the EU would be rather weak. There is no doubt, however, that the UK will seek to get favorable trade deals with ASEAN countries so that a new UK-EU rivalry will characterize EU-ASEAN relations in the future. As regards the UK’s international reputation, there is a negative development in the course of the corona epidemic which shows that the UK’s health system and the government’s health policy are rather weak: More than 40,000 COVID-19 deaths are a shocking indicator, compared to the 9000 COVID-19 deaths in Germany in mid-June 2020; other EU countries such as Italy, Spain and Belgium – each with rather weak coalition governments – also show high COVID-19 fatality ratios. If the political reaction pattern identified by Aksoy et al. (2020) for the case of epidemic shocks holds in the coronavirus pandemic, there will be negative political scars for about two decades in those (and other) countries and possibly a weakening of democracy. What will be the effects on the EU as well as the US, China, ASEAN and other countries, how will international cooperation in key policy fields be affected?

**Fig. 1:**
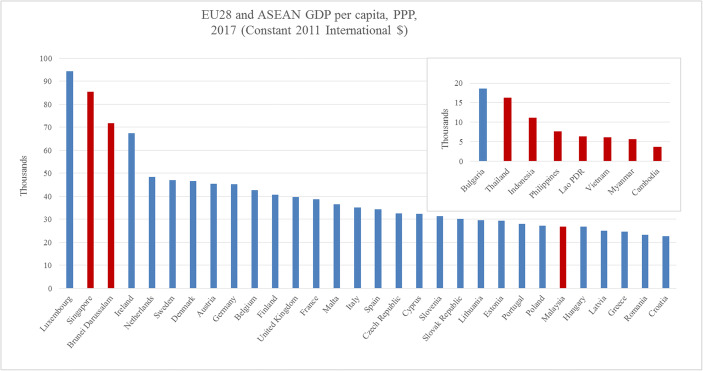
Per Capita Income in the EU Countries and in Selected ASEAN Countries. *Source: own representation of data from the World Development Indicators*

**Fig. 2:**
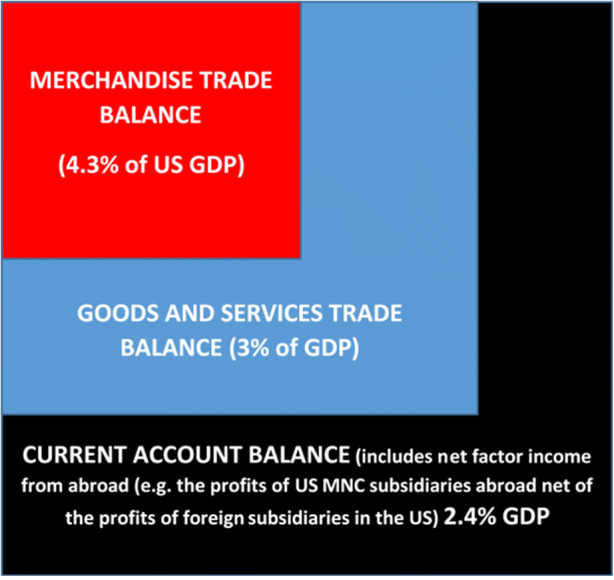
Trump Complaint Box. *Source: Own representation using data from the Bureau of Economic Analysis, Preliminary Estimates of U.S. International Transactions, March 2019*

In economic terms, many ASEAN countries may be expected to come close to the per capita income position of the eastern European EU countries by 2030. It is interesting to note that several ASEAN countries have achieved per capita income positions that are close to or above that of Bulgaria and Romania in 2018 (see Fig. 1 and 2). Narrowing per capita income differences imply better prospects for political cooperation as political preferences become more similar.

### Historical watershed year 2016

2016 represents an historical watershed moment for the Western world as the EU faced the first rejection of membership from an existing EU country, namely the UK, with its majority in a referendum for BREXIT, and since the US has voted for a populist president – Donald Trump - who has started to change the global institutional setting and to embark upon an aggressive trade policy soon after he had already refused to sign the Trans-Pacific Partnership (TPP; negotiations concluded under President Obama) with eleven partner countries, including Australia and Japan. Trump also did not continue negotiations on the Transatlantic Trade and Investment Partnership (TTIP) which was a US-EU deep integration project for more transatlantic free trade – with a strong focus also on an international investment treaty. As regards China, the Trump Administration is particularly dissatisfied since there are issues both in the field of trade as well as in the area of foreign direct investment (FDI) and intellectual property rights.

The demise of the socialist systems in Eastern Europe in the late 1990s and the disintegration of the Soviet Union in 1991 initially contributed to a reinforcement of the World Market Economy, not least since China and the then new Russia joined the World Trade Organization and due to the process of EU Eastern enlargement and ASEAN integration deepening, while the TTIP and TPP negotiations seemed to indicate a stronger regional integration and global integration dynamics. However, the declining external pressure on the EU after the collapse of the Soviet Union and the imperfect monetary integration in the EU have brought about a weakening of EU integration and this, along with the rise of China as the world’s No. 1 exporter, has destabilized EU integration as well as the US whose expansion was dampened by the Eurozone crisis. The latter also faced a massive decline of the income share of the lower half of the US income pyramid since the 1980s – this can be shown to be a driver of US structural populism which means that even if President Trump should not be re-elected in November 2020, there remains significant pressure in the US for political populism which is a totally new Western world and which certainly presents a major challenge for Europe: Trust in US politico-economic stability could decline dramatically in the decade after 2016. Issues of political risk – so far familiar from some Latin American countries – could become a regular issue in Western OECD countries and this could include EU countries and the UK, as will be argued subsequently.

Trump’s presidential election victory owes much to a high share of votes from relatively poor households and unskilled workers, respectively. For decades, the latter could earn relatively high wages in the manufacturing industry, but with high productivity growth and a rather slow growth of domestic demand for industrial goods, the share of workers with no college degree in overall manufacturing employment in the US has clearly declined over time (Lawrence 2017). China’s rising exports to OECD countries might have contributed to mounting dissatisfaction among workers in both the USA and the UK since the export of Chinese industrial goods contributes to wage polarization there as it does in many other industrialized countries (see the OECD study by Breemersch et al. 2017).

The economic relations of the US with China again played a major role in the corona crisis as the coronavirus pandemic seems to have started in the Chinese province of Hubei in late 2019. As a by-product of the international epidemic, the Corona World Recession emerged in 2020 and this also caused conflicts with China at the World Health Organization (WHO) - which President Trump announced the US would leave in late May 2020 so that another cornerstone of multilateralism was about be massively weakened; in this case by the largest financial contributor amongst member countries, namely the United States. The WHO is an unusual organization since only about 20% of its budget comes from member countries’ standard contributions, the rest is from private foundations as well as additional contributions by various member states; in the case of China, the Chinese government had announced in 2020 that China’s contribution would be raised by a one-off increase of two billion dollars. The Trump Administration had argued that the US’ poor response in the Corona Crisis was largely due to China not communicating early on its insights about a new epidemic in late 2019 – and that the WHO had been too uncritical of China, indeed the WHO pointed out that the Chinese epidemic policy was a great success story.

In the decades since 1995, the world economy has moved towards more economic integration and in parallel – since about 1985 when foreign direct investment has increased strongly – towards more economic globalization. As China had opened up in 1978, Asian economic dynamics strongly increased. It is remarkable that the rise of the Newly Industrializing Countries (NICs: Taiwan, Republic of Korea and Hong Kong plus some ASEAN countries, prominently including Singapore) indeed had contributed to the crisis of socialist Eastern European countries which were forced to export higher volumes of export goods - in a context of more competitive international markets and rising high quality exports from NICs – in order to get the required volume of import goods in the 1980s (Welfens 1992). Since the collapse of the Soviet Union and its bloc in Eastern Europe, the Western world has expanded as a globalizing market economy: 29 transforming economies have integrated into the world economy and finally Russia became a member country of the World Trade Organizations in 2012, eleven years after China’s membership.

Until 2016, the world economy seemed to make progress with further trade and investment liberalization; the EU and the United States, for example, wanted to conclude a Transatlantic Trade and Investment Partnership (TTIP) under the Administration of President Obama, but this project could not be finished; to same extent due to the lackluster commitment of Germany and France both of which did not attach strategic value to this project – governments in both countries faced some resistance against the envisaged TTIP in parts of society, but the government in Germany was particularly hesitant to explain the benefits for Germany and the EU, respectively; the investment protection issue was also greatly emphasized by opponents in several EU countries. Meanwhile the Obama Administration had concluded the Trans-Pacific-Partnership (TPP) agreement which had even brought Japan on board in the final stages of negotiations. At least – with many political problems faced before the conclusion of the treaty - the EU was able to conclude a partnership agreement with Canada (“CETA”) which later became a potential benchmark for the question of how the EU27 and the UK, leaving on January 31st 2020, on the basis of the 2016 British EU referendum, could organize their future relations.

With the British EU referendum of 2016, which rather surprisingly brought a majority for BREXIT and the election of the populist US President Trump in November of that year, the Western-led era of globalization seems to have come to a pause.

To the extent that BREXIT means less liberal trade between the UK and the EU27 after the transition period until the end of 2020 – until then the UK remains in the EU single market – and to the extent that the Trump Administration is clearly pursuing a rather protectionist trade policy agenda, the two historically leading Western countries in terms of free trade have changed their position dramatically. Given the considerable EU-UK integration benefits, achieved over more than 46 years of EU membership, one might consider BREXIT to be a rather strange decision in economic terms; and it is so indeed, if one follows the arguments spelled out the Treasury’s report on British benefits of EU membership (HM Government 2016) which was published in April 2016 and suggested that a hard BREXIT – the UK leaving the EU without a free trade agreement – could bring an economic loss of 7 to 10% of GDP. However, the old economic and political elites, nearly all of whom had recommended Remain as a decision in the referendum, had lost much credibility in both the US and the UK in the years of the Transatlantic Banking Crisis 2008/09 which was marked by a sharp economic output contraction in 2009 in Western OECD countries and clearly showed a new vulnerability of both the US and UK as well as some other EU countries. The Euro Crisis of 2010–2014 – involving mainly Greece, Ireland, Portugal, Cyprus and at some point Spain – brought a rather slow economic recovery in the Eurozone where Italy’s growth problems were exacerbated in the context of that crisis and even a populist coalition government which came to power in 2018 (the first Conte government of 2018/19 had two populist parties on board, the second Conte government of 2019 still had the left-wing MoVimento 5 Stelle Party in government while the right-wing Lega Party had left the coalition); Italy could be understood almost as a first political warning signal concerning US spillovers following the election of the populist President Trump in late 2016. Populism means nationalism and protectionism and therefore undermines regional integration and economic globalization (Welfens 2019a). A populist president in the US is a shock to the world economy.

### Overlapping crisis dynamics

The US had turned its economic attention to China and Asia since the beginning of the twenty-first century and President Obama was indeed offering TTIP as a political project to the EU because EU countries felt disappointed about the US push for TTP and thus more emphasis on Asian-US economic relations. Obama’s successor, Donald Trump, adopted a populist political and trade policy agenda and decided to pull the US out from TTP leaving Japan in a surprising lead situation for a country which had itself only joined the negotiation process rather late. The reason for Trump’s political success in the presidential election of 2016, coming also as a surprise for most observers in the US at that time, brought a radical change for the US and its Western allies: The EU countries no longer received political support from the US and indeed witnessed Trump publicly supporting the BREXIT project. From a standard economic perspective, BREXIT was bound to economically weaken both its NATO ally UK and the large group of NATO member countries in the EU27; this normally would not be in the interest of the United States, but the populist President Trump ignored standard economic wisdom and indeed often did not want to listen to his leading experts; Kevin Hassett, for example, left his position as the chief of the Council of Economic Advisors in 2019. Trump’s Administration also embarked upon an ongoing US-Sino trade conflict and Trump even sought conflicts with European countries which had been traditional partners: by pulling out of the TTIP negotiations and by imposing import tariffs for aluminum and steel as well as other products. Moreover, the Trump Administration suffered from the particular problem of a lack of competence as only three-quarters of vacant positions left by political appointees of the Obama Administration had been filled by Trump. This makes it almost impossible to coordinate the US and its western allies in the situation of an international economic crisis – the IMF, for example, would not really know with whom they should talk in the Treasury in such a situation. This, however, was exactly the situation which occurred in 2020 with the corona shocks: Affecting the health systems and the economic systems of almost 200 countries worldwide. While China was the origin of the coronavirus pandemic, the countries worst affected were countries in Europe, North America and Latin America.

The question arises as to how the corona shocks will affect the EU and the ASEAN countries in this broader context, namely in the context of a parallel rise of the US-Sino politico-economic relations. Moreover, how will the corona shocks affect the EU and to what extent is the overlap with BREXIT dynamics and Trump’s aggressive trade policy a problem for regional integration in Europe and Asia? Finally, what are the implications of BREXIT for EU-ASEAN economic relations and what could replace waning US leadership – such leadership in international economic crises was always visible in the decades after 1945; until 2020 when the Corona World Recession called for just such US leadership while the populist US president decided not to demonstrate any. This, in turn, raises in passing the question as to what the reason for the rise of populism in the US actually is: If there is only a short-term rise of political populism, people in the Western world, the OECD group and in ASEAN and China will not have to worry much about a transitory political shift in the United States, but if Trump represents a structural populist challenge, the situation is much more difficult for the US partner countries and for the global economy at large.

The following analysis looks at first at US trade policy (Section 2), before taking a brief look at the BREXIT challenges (Section 3) and the problems of the corona shocks and a potential EU crisis (Section 4). Section 5 offers some key policy conclusions and suggests that there could be a considerable change in global governance in the post-corona setting. Multilateralism seems to be seriously weakened and it is as yet unclear to what extent the option, for example, of a networked leadership – involving the EU, ASEAN and Mercosur (plus X) – would be able to replace US leadership. Even if Trump’s successor should switch back to a more traditional US policy approach, the structural nature of US populism suggests that the traditional Western stability and global leadership can hardly be restored. Post-corona, the OECD countries could face much more political risk than in previous decades, weaker trust in democratic western institutions and, as a consequence, lower economic growth which in turn could encourage the rise of populism in some western countries; the latter would ultimately undermine multilateralism in a very serious way. With the Trump Administration blocking the Nord Stream II gas pipeline link between Russia and Germany in 2019 new trade conflicts emerge and high stranded investment costs could become a problem for the firms involved which implies new barriers for growth and efficiency gains – a conflict which should have been avoided and shows a lack of multilateral rules for international investment projects. At the same time the Corona EU recovery program with emphasis on costly green innovations such as hydrogen used in steel production could bring new trade conflicts to the extent that the EU could impose new tariffs on “dirty” steel imports in the future; climate policy initiatives matter for trade policy.

## Trade policy perspectives under the trump administration

### International trade conflicts and Corona shocks

President Trump has promised to bring industrial jobs back to the US and he has indicated that to this end tariff protectionism could be useful and will be applied. After a first presidential year which saw a tax reform in 2017 – reducing income tax rates as well as corporate tax rates in the US – the Trump Administration has started to push for more public investment, including the building of a partly new US-Mexican border wall. The aggressive trade policy of Trump implemented in 2018/2019 is difficult to understand since the metal import tariffs on aluminum and steel, justified on the grounds of national security by Trump, concern in more than 80% of the export volume NATO allies, including Canada, the UK, Germany and France. There really is a risk of a global trade war as President Trump has also indicated the option of imposing higher import tariffs on automobiles. Most countries facing the US import tariffs on aluminum and steel have indicated their willingness to impose counter-balancing tariffs on US imports. The EU as well as Canada and Mexico have made a complaint against the US at the World Trade Organization for which a US import tariff based on national security reasons is a rather rare case of conflict. The problem is that the WTO is no longer operational in the field of dispute settlement as from November 2019 the WTO will no longer be operational in the field of dispute settlement since the necessary election of new judges to fill vacant places at the appellate body could not take place in 2018 as this was blocked by the Trump Administration. Hence, the multilateral system which is based on International Organizations in various fields and US political leadership supporting these organizations is endangered.

The US refusal to allow the election of new judges to the World Trade Organization’s appellate body in 2019 brought about a stop of the standard WTO dispute settlement mechanism and thereby weakened the prospects for both free trade and efficient global foreign direct investment – subsidiaries abroad would like to be sure that exports and imports can be made without restrictions. Moreover, Trump had announced his decision to withdraw the US from the UN Paris Accord regarding climate change, which, of course, partly soured US-French relations and US-EU relations as well (the actual withdrawal from the Paris Accord takes effect on November 4, 2020). Thus, the populist US president has strongly undermined key fields of international cooperation, and by weakening International Organizations he has indeed implemented part of the traditional agenda of the Republican Party.

As regards the US trade balance deficit US President Trump has repeatedly complained in 2017–19 that the trade balance deficits were too high. However, the US trade balance deficit hardly can be considered to be a relevant macroeconomic measure unless the US would face considerable unemployment which was not the case in 2018/2019. At first glance a trade balance deficit-GDP ratio of 4.3% in 2018 is rather high (see the subsequent graph), but the current account balance is more relevant in economic terms as it will determine the evolution of the US foreign indebtedness. Since the US has a surplus in the international services balance already the US balance on goods and services was only 3% of GDP in 2018; taking into account also net factor income from abroad and unilateral transfers gives a current account-GDP ratio of 2.4%. If the US has a long run real GDP growth rate of 2% the long run foreign indebtedness-GDP ratio will lead to a steady state value of that ratio of 125% if the current account-GDP ratio stays at 2.4%. In an international environment of low interest rates, this situation is not dramatic for the US since a real interest rate of 1%, for example, would imply net interest payments of 1.25% of GDP to creditors abroad.

There could be, however, a more long-term problem in the sense that an ongoing expansionary fiscal policy of President Trump in 2018/19 is likely to reinforce both the US government deficit-GDP ratio and the current account deficit-GDP ratio. As regards transatlantic trade relations between the US and the EU, one may anticipate that President Trump will put enhanced pressure on EU countries to buy more liquid natural gas as well as more military equipment from the US. It seems that here, political pressure from the US President vis-à-vis EU countries is relevant. At the same time, it is remarkable that in 2018, the US Council of Economic Advisors argued that Scandinavian EU countries in 2016 were far behind the US in terms of per capita consumption figures, but these arguments are quite inaccurate if one considers effective lifetime per capita consumption; Norway, for example, is no longer 18 points behinds the US as argued in the paper of the Council of Economic Advisers (2018b), rather an adequate lifetime perspective shows that Norway is in fact slightly ahead of the US and if one compares the US lifetime effective per capita GDP with that of Germany and France – where the populations enjoy higher life expectancy than in the United States – the figure for the three countries (with real per capita GDP growth rates in all three countries assumed to be identical in the long run) are equal as the subsequent Table 1 shows. Effective per capita lifetime income means that this income category is net of health care expenditures which relative to GDP is 1/3 higher in the US than in Germany and France; in addition, the relevant comparisons (Baier and Welfens 2019) take into account that West European countries have more leisure time and higher – paid – vacations than US workers.

**Table 1:** Effective* Disposable Nominal Income (y’; yearly data) of Germany + France Relative to the US, 1995–2015, (‘000 US $ Purchasing Power Parity (PPP))

	**1995**	**2000**	**2005**	**2010**	**2015**	**Life Expectancy (L‘)**	**L’ x y‘**
France	14,244	16,741	19,549	22,909	24,576	82.4	*2,025,056*
Germany	15,221	17,894	19,643	23,580	25,855	81.1	*2,096,881*
United States	15,706	19,639	22,154	23,826	26,302	78.6	*2,067,298*
Average Difference; in percent (FR + DE/US)	6	12	12	2	4		

*Note: Here, “effective” means corrected for transatlantic differences in holiday time and health care expenditures: For Germany and France, annual nominal income has been multiplied by 1.1 to reflect a month of extra holiday in these countries, compared to the US; the official US figures have been reduced by 18% (expected US health care expenditures relative to GDP) and those of Germany and France by 11% (health care expenditures relative to GDP in France and Germany in 2017). The last column multiplies the 2015 annual effective income with life expectancy; this overestimates somewhat the EU advantage and the lead of Germany and France, respectively, since future income should in normal circumstances be discounted by some adequate discount factor

Source: WELFENS (2019a) Table 2.3, p. 54; EIIW calculations using data available from the OECD Income Distribution Database

In a 2011 publication on macroeconomic imbalances, the ECB (2018) has argued that the US is a kind of a natural policy benchmark to achieve – obviously assuming that the US is leading vis-à-vis Western Europe -, but the implicit conclusion of the ECB that all EU countries would be wise to adopt the same deregulations and other institutional settings as the US seems to be quite doubtful. One may add that the relative weakness of the US health system compared to that of Germany became rather obvious in the corona crisis of 2020 (Welfens 2020a); the Global Health Security Index is not very convincing with a global US lead in nearly all sub-indicators of the overall index published for the first time in 2019 (NTI/JOHNS HOPKINS UNIVERSITY 2019).

There is a remarkable lack of skilled staff in the Treasury and the Department of Commerce. There are at least 1000 expert positions which had been occupied by appointees of the Obama Administration which have not been filled under President Trump which is not only leading to a poor and inconsistent trade policy on the part of the US but also implies that the US is hardly in a position to be able to provide international leadership in the Corona World Recession. Trump has replaced only three-quarters of the political appointees of the Obama Administration. Secondly, the trade conflicts are likely to become worse in the medium term since the Trump Administration has adopted an economic policy (higher military expenditures and an aggressive tax reform in 2018/19) which widens the US current account and will also raise the trade balance deficit – President Trump should be expected to become more aggressive vis-à-vis the EU and China in the field of trade policy; the corona recession dampens transitorily the transatlantic US current account deficit.

With already high deficit-GDP ratios in the US in an economic upswing, it is clear that the corona recession brings about a record deficit-GDP ratio; for 2020, the IMF (2020b) shows 15% in its Fiscal Monitor analysis of mid-2020, but with an additional stabilization fiscal package, the US deficit-GDP ratio could move to nearly 19% of GDP in 2020. If one assumes that the deficit-GDP ratio is not reduced by more than 3 points per year, the US-debt GDP ratio would increase from 100% in 2019 to about 160% in 2025 so that the traditional AAA rating of the US would be no longer applicable at all leading rating agencies (S&P had already reduced the US sovereign rating to AA after the banking crisis). Such an adjustment is likely to bring more downgradings for OECD countries, including for Italy in the Eurozone and, outside the EU, for the post-BREXIT UK.

Post-BREXIT UK will face a much lower growth rate than in the decade before 2016 and therefore will adopt lower corporate taxation and financial deregulation. As the USA has already also added banking deregulation under Trump, the joint UK-US banking deregulation is likely to bring about the next European deregulation wave and hence also the next transatlantic banking crisis in the medium term (within a decade or so). There is a high risk that the US under populist presidents will bring about the end of the age of multilateralism and will push for a new Great Power Regime that will be similar to the late nineteenth century system in Europe, however, this time with new actors such as China, Japan and India; and the countries of the EU will, once disbanded, have to decide whether they want to be vassals of the US, Russia or China; for the moment this is only a scenario. The existing multilateral system and thus the role of the International Rule of Law for international trade and investment can only be preserved if the EU27 and ASEAN plus China would cooperate more strongly in the future. EU reforms are necessary for a long-term stabilization of the EU; however, the necessary reforms are not very likely to be taken in a timely fashion and one therefore cannot rule out that the world economy will be shaped by regional disintegration, weaker globalization, the end of multilateralism and a lasting rise of populism in the Western world in the early twenty-first century: Reduced economic growth and lower prosperity as well as more economic instability would be the key result. Surprisingly, some recent Council of Economic Advisers’ studies have a rather strong ideological bias. The Economic Report of the President (Council of Economic Advisers 2018a) has a view on trade which was not in line with state-of-the-art analysis and the CEA (2018b) paper on “The Opportunity Cost of Socialism” is a strange study which not only criticizes socialism in Cuba and Venezuela but seems to suggest that Nordic European countries are socialist countries and lag economically much behind the US – both conjectures are simply wrong and undermine the reputation of the Council of Economic Advisers. As regards regional integration in Asia the Trump Administration has pulled out of TPP and thus has left trade liberalization to Asian countries as well as other countries.

### Asian perspectives and EU aspects

Since 2011, ASEAN countries have negotiated with China, Japan, the Republic of Korea, Australia and New Zealand plus India about a Regional Economic Comprehensive Partnership project; but in 2019 India opted out of RECP – mainly because the country is afraid of facing stronger competition from China if India would be part of RECP. In any case, since 2011 Asia has become the region with most progress in regional integration dynamics for at least a decade. If one considers the countries involved, this could contribute to stronger economic growth in Asia. Whether or not catching-up economies could face problems of a middle-income trap (Wagner 2017) is unclear. At least as regards China, one may argue that this country is large and internationally influential enough that it should be able to avoid such a trap on the one hand; on the other hand, there is the political conflict about the political regime in Hong Kong and the strong US-Sino trade conflict since 2017 which stand for particular risk involving serious international political conflicts that in turn could strongly undermine China’s export and trade dynamics, respectively. While China’s one belt one road (OBOR; i.e., the new silk roads initiative) approach envisages enhanced trade and foreign direct investment relations with more than 50 countries in Asia and Europe, one may argue that the OBOR approach is a very long-term project which will be realized only gradually. It still is unclear how the corona shocks will affect Asia, the EU and the Americas in the medium term, but apart from China, as the initial starting point of the coronavirus pandemic, few countries in Asia seem to be affected strongly. If one compares the corona fatality ratios (Table 2) in the EU and ASEAN, it is noteworthy that only the Philippines and Indonesia are ASEAN countries with relatively strong exposure to the corona shocks, namely with ratios which are above the relevant figure for Slovakia (the EU27 country with the lowest fatality ratio). Moreover, the IMF’s projected output decline for ASEAN countries mostly are much smaller than for EU countries 2020 (Table 3).

**Table 2:** COVID-19 Fatality ratio of EU, ASEAN, and China (cumulated COVID-19 fatalities to June 2, 2020, per million population in 2018)

*Rank*	*Country*	*Fatalities per million*	*Rank*	*Country*	*Fatalities per million*	*Rank*	*Country*	*Fatalities per million*
1	Belgium	818.49	14	Romania	66.48	27	Latvia	12.72
2	Spain	597.59	15	Finland	57.75	28	Philippines	8.76
3	United Kingdom	575.16	16	Hungary	55.07	29	Indonesia	6.00
4	Italy	553.66	17	Slovenia	51.95	30	Slovakia	5.13
5	France	441.73	18	Estonia	51.26	31	Brunei	4.57
6	Sweden	435.97	19	Czech Republic	29.98	32	Singapore	4.10
7	Netherlands	347.95	20	Poland	28.38	33	Malaysia	3.55
8	Ireland	334.16	21	Lithuania	25.71	34	China	3.22
9	Luxembourg	175.73	22	Croatia	25.09	35	Thailand	0.82
10	Portugal	139.65	23	Bulgaria	20.72	36	Myanmar	0.11
11	Germany	101.71	24	Malta	20.38	37	Cambodia	0.00
12	Denmark	99.44	25	Cyprus	19.41	38	Laos	0.00
13	Austria	74.17	26	Greece	16.79	39	Vietnam	0.00

Source: Own representation of data available from Our World in Data (2020)

**Table 3:** Forecasted Real GDP Growth of Selected Countries (IMF 2020a)

Country	2019	2020	2021	Country	2019	2020	2021
Greece	1.90	−10.00	5.10	Thailand	2.40	−6.70	6.10
Italy	0.30	−9.10	4.80	Singapore	0.70	−3.50	3.00
Croatia	2.90	−9.00	4.90	Malaysia	4.30	−1.70	9.00
Latvia	2.20	−8.60	8.30	Cambodia	7.00	−1.60	6.10
Lithuania	3.90	−8.10	8.20	**ASEAN-5**	**4.80**	**−0.60**	**7.80**
Portugal	2.20	−8.00	5.00	Indonesia	5.00	0.50	8.20
Slovenia	2.40	−8.00	5.40	Philippines	5.90	0.60	7.60
Spain	2.00	−8.00	4.30	Lao P.D.R.	4.70	0.70	5.60
Estonia	4.30	−7.50	7.90	Brunei Darussalam	3.90	1.30	3.50
Netherlands	1.80	−7.50	3.00	Myanmar	6.50	1.80	7.50
Eurozone	**1.20**	**−7.50**	**4.70**	Vietnam	7.00	2.70	7.00
France	1.30	−7.20	4.50				
EU	**1.70**	**−7.10**	**4.80**				
Austria	1.60	−7.00	4.50				
Germany	0.60	−7.00	5.20				
Belgium	1.40	−6.90	4.60				
Ireland	5.50	−6.80	6.30				
Sweden	1.20	−6.80	5.20				
Cyprus	3.20	−6.50	5.60				
Czechia	2.60	−6.50	7.50				
Denmark	2.40	−6.50	6.00				
Slovak Rep.	2.30	−6.20	5.00				
Finland	1.00	−6.00	3.10				
Romania	4.10	−5.00	3.90				
Luxembourg	2.30	−4.90	4.80				
Poland	4.10	−4.60	4.20				
Hungary	4.90	−3.10	4.20				
Malta	4.40	−2.80	7.00				

Source: Own representation based on data available from the IMF (2020a), World Economic Outlook, April 2020; countries ranked according to forecasted output decline in 2020

**Table 4:**
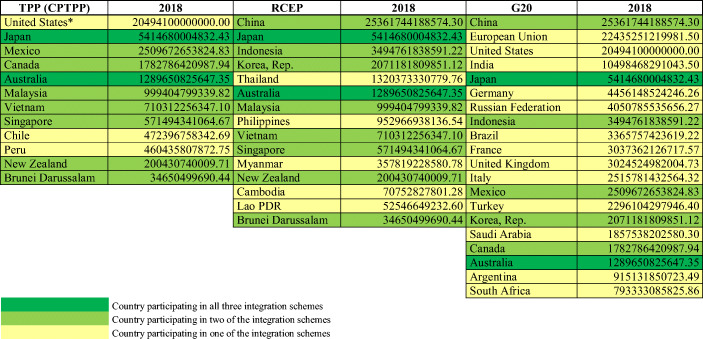
TPP, RCEP (ASEAN + FTA partners China, Japan, Republic of Korea, Australia, New Zealand**) and G20 – Member/signatory countries ranked by GDP, PPP in Current International Dollars, 2018

*Note: The United States later withdrew from the TPP negotiations and agreement after a presidential memorandum to the US Trade Representative on January 23, 2017 (Trump Administration). The other 11 signatories of TPP proceeded with signing the Comprehensive and Progressive Agreement for Trans-Pacific Partnership (CPTPP) on 8 March 2018. It has since been ratified and entered into force for 7 of the 11 signatories. ** India has opted out of the Regional Comprehensive Economic Partnership (RECP) in 2019; RECP represents about 30% of the world population and 30% of the world national income. The RECP initiative goes back to 2011: at the 19th ASEAN Summit (Nov. 14–19) RCEP was suggested where Japan was one of the driving countries. During the 19th ASEAN Summit held 14–19 November 2011, the Regional Comprehensive Economic Partnership (RCEP) was launched as a regional integration project in Asia – in August a joint initiative of China and Japan for a broader regional integration treaty had been welcomed by East Asian summit countries

Among the ASEAN countries, Indonesia is the most populous country and has shown high levels of ambition in following a broader free trade policy, including negotiations about a Free Trade Area with Canada (the Indonesian government relies on CGE modelling as a crucial element for assessing potential benefits from such an FTA). It seems obvious that the influence of China is increasing in Asia through the RECP project, but it should not be overlooked that a comparison of the membership of TPP (renamed the Comprehensive and Progressive Agreement for Trans-Pacific Partnership (CPTPP) after the US pulled out in 2017 under the Trump Administration), RECP and the G20 membership indicates a strong role of Japan and Australia which are both represented in all three multilateral institutions. Japan which long has been rather inactive in regional integration schemes has become a regional integration leader due to the pulling out of the US from the TPP project which reinforces Japan’s multilateralism. For China, the RECP project would be a first regional integration scheme and could be seen as part of first broader steps of China’s government towards multilateralism which started in 2001 with the accession to the World Trade Organization and in 2014 the creation of the multilateral Asian Infrastructure Investment Bank (AIIB). While some EU countries were founding member countries of the AIIB, including Germany, France and the UK, Japan and the US did not want to join the AIIB, rather both countries want to defend the strong regional multilateral role of the Asian Development Bank (ADB) which is traditionally led by a representative from Japan. Given the globally leading role of China, there is strong interest of both ASEAN and EU countries to firmly anchor China in a pro-multilateral policy orientation. However, the strong US-Sino trade conflicts and the undermining of the World Trade Organization by President Trump means that the global strength of multilateralism has not been strengthened in a reliable way. The US policy stance is quite crucial and a future populist president is just as unlikely to support multilateralism. Both the US and China could decide to pursue bilateralism as both countries are really large economies and could decide to organize a system of international economic relations through by-passing international organizations.

### Economic globalization, inequality and the rise of populism in the US

The reasons behind Donald Trump’s rather surprising election victory are manifold. While it is true that Trump has obtained votes from all strata of the economy, it is also clear that his rival Hillary Clinton obtained a rather low share of votes among those with an income level of less than $ 30,000 (BBC 2016): Obama had 63% support from this group compared with 35% of votes for Mitt Romney – Hillary Clinton achieved 53% support, Donald Trump 41%; 51% of voters with a high school diploma supported Donald Trump, while Hillary Clinton obtained 45% support. Voters with a high school diploma might have been concerned about the risk of declining income shares for the median household (or the lower 50% of the income pyramid) in the US. While per capita income convergence could be observed across countries in the three decades after 1980, income inequality within many countries has increased (it is interesting to note that the rising income share of the top 1% of income in Switzerland (Föllmi and Martinez 2017) mainly stems from those rich individuals who have income accruing from various international sources and thus the globalization of industry via international investment – and rather inefficient taxation of profits - could play a crucial role for rising income inequality within OECD countries; with the US being a leader in outward foreign direct investment). Rather inefficient taxation of international capital income could play a particular role for rising inequality in both the US and some other OECD countries.

**Fig. 3:**
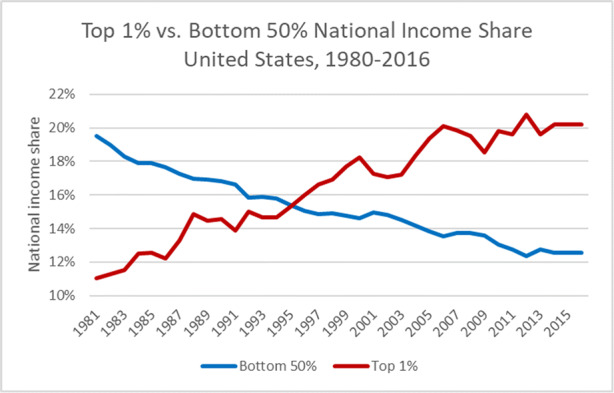
Top 1% vs. Bottom 50% National Income Shares US (1980–2016). Source: Own representation based on data available from the World Inequality Database http://www.wid.world

**Fig. 4:**
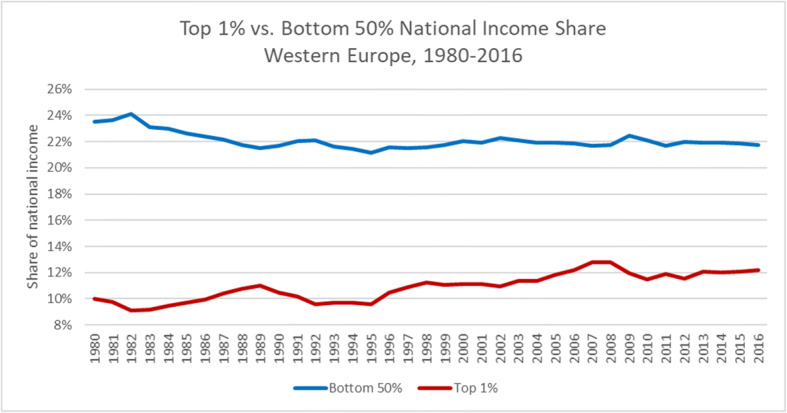
Top 1% vs. Bottom 50% National Income Shares Western Europe (1980–2016). Source: Own representation based on data available from the World Inequality Database http://www.wid.world

The US has faced a long-term increase of inequality as a consequence of structural changes in the US and economic globalization. One key aspect to understand the victory of Trump is the fact that American society has faced a long-term decline of the income share of the lower half of the US income pyramid. The share of this group has declined from 21% in 1980 to 13% in 2016 as has been shown by (Alvaredo et al. 2018, see Figs. 3 and 4 below) – a much more dramatic decline than in Western Europe where the respective income share in 1980 was 24% and around 22% in 2016; the share of the upper 10 % of China and India, respectively, has strongly increased in the period 1980–2016 (China seems to approach the US income inequality position in the medium term).

**Table 5:** Banzhaf Power Index for EU27 Countries Pre- and Post-BREXIT

	EU with UK		EU without UK	
	**Population (%)**	**Banzhaf Index (%)**	**Population (%)**	**Banzhaf Index (%)**
Germany	15.90	10.20	18.30	11.90
France	13.00	8.40	14.90	9.90
Italy	12.00	7.90	13.70	9.20
Spain	9.20	6.20	10.50	7.70
Poland	7.50	5.20	8.60	6.60
Romania	3.90	3.80	4.50	4.00
Netherlands	3.30	3.50	3.80	3.70
Belgium	2.20	2.90	2.50	3.00
Greece	2.20	2.90	2.50	3.00
Czechia	2.10	2.80	2.40	2.90
Portugal	2.10	2.80	2.40	2.90
Hungary	1.90	2.80	2.20	2.90
Sweden	1.90	2.70	2.20	2.80
Austria	1.70	2.60	1.90	2.70
Bulgaria	1.40	2.50	1.60	2.50
Denmark	1.10	2.30	1.30	2.30
Finland	1.10	2.30	1.20	2.30
Slovak Rep.	1.10	2.30	1.20	2.30
Ireland	0.90	2.20	1.00	2.20
Croatia	0.80	2.20	1.00	2.20
Lithuania	0.60	2.00	0.70	2.00
Slovenia	0.40	2.00	0.50	1.90
Latvia	0.40	2.00	0.50	1.90
Estonia	0.30	1.90	0.30	1.80
Cyprus	0.20	1.80	0.20	1.80
Luxembourg	0.10	1.80	0.10	1.70
Malta	0.10	1.80	0.10	1.70

Source: Own representation based on Kirsch (2016), Table 1

Trump has emphasized the role of the “forgotten men and women” in the US which effectively referred to the lower half of the income pyramid. It is not fully clear why the decline of the lower half of the US income was so dramatic over time. The massive income tax cuts under the Reagan Administration as well as globalization and the expansion of the digital economy – with “superstar firms” and new oligopolies - might have played a considerable role in rising US inequality. Trump’s tax reform of 2017 has also reinforced economic inequality so that his policy is not really aimed at helping the forgotten men and women; but it is true that Trump’s strangely expansionary fiscal policy in the middle of the economic upswing 2017–2019 has reduced the unemployment rate considerably (in 2017/18, the employment rate did not increase much, but a positive development became visible in 2019). Nevertheless, the US remains a Western outlier with its enormous increase in inequality in the period between 1981 and 2016 and the fact that voters do not seem to expect the US government to adopt an economic policy aimed at reducing this rise in inequality could imply that there is a structural US populism as voters from the lower strata – expecting instead that big companies would adopt measures to reduce wage inequality at the firm level – will be frustrated time and again. US multinationals will not start to cut the salaries of its top management. Moreover, the corona shock could raise the share of the digital economy in which imperfect competition and a higher salary share for skilled workers are typical so that the skilled wage premium in the US could further increase.

The rise of populism in the US is understood here as reflecting the combination of rising poverty in the US – actually combined with rather modest life expectancy compared to Western Europe (see appendix) – and a digital upheaval by the lower strata of society which find in the internet a cheap platform to get organized and to get politically framed by such a digitally active candidate as Donald Trump. The internet user density in the US had reached 76% in 2016 so that almost all voters had some internet access. Search engine technology made it easier for presidential candidates to target specific voter groups and Trump’s deal with the British company Cambridge Analytica was obviously quite useful for his campaign. In the internet age, emotionalized campaigns have become easier and less costly than traditional political campaigns and the propensity of users to consume information and news in a rather superfluous way from many digital platforms could reduce the quality of knowledge in critical fields in the overall economy; not least because experts’ influence or the impact of scientific think tanks seem to have reduced (ultimately, representatives of authoritarian democracy models might find it rather simple to control larger strata of voters so that political competition and democracy, respectively, might be impaired). The explanation of Trump’s election is not easy at first sight, however, taking the results from survey results and economic analysis together, one may offer the following compact view (the following draws on insights from a UN workshop in 2018):

- The declining income share of the lower half of the US households in the income pyramid is a serious problem. If the lower 50% of income earners have only 13% of national income and are heading towards about 10% in 2025 – down from 21% in 1980 – this must contribute to massive frustration. This holds all the more as the majority of US voters hold the view that hard work is the basis for upward economic mobility and success (Lindh and McCall 2018); the survey results of Lindh/McCall also show an interesting result, namely that the US voters are concerned about the rise of economic inequality and that they consider as an adequate remedy not so much economic policy intervention, but rather that multinational companies should adopt measures that would reduce inequality – this, however, is a totally illusory view and bound to create further voter frustration in the US. The contradictions of expectations and reality will thus continue for a majority of US voters which in turn will enhance populist political forces in the US.
- During the presidential campaign, Donald Trump emphasized that social policies should not be reinforced. In his Phoenix speech, he argued that social policies would mainly be exploited by illegal immigrants and that this should be the basis for fighting immigration as well as social policies (Eichengreen 2018, p.112). Trump’s argument was that social policies are not really needed in the US, but a comparison of Germany, France and the Scandinavian countries in the corona shock setting of 2020 suggests that Western EU countries are better able to absorb the corona recession than the US where about 40 million workers had lost their job in the first half of 2020 with about three-quarters of those also medical insurance as health insurance is typically linked to having a job in the US. The random stress test “corona shock” has exposed many weaknesses of economic systems as well as partly inadequate epidemic policies; for the US, poor results imply politico-economic destabilization. This is not only bad for the US population, it is also a problem for foreign investors – more than one quarter of global FDI stocks are in the United States. Political instability in the UK and the US is a rather new phenomenon since 2016 and economic theory suggests that lower growth should be expected in such a setting in the medium and long run. Subsequently, it will also be pointed out that the corona pandemic shock is likely to have broadly undermined, trust particularly that of young people, in institutions and democracy in OECD countries and some other countries as well.

It is rather remarkable that many of the strange conjectures of Trump were taken seriously in broad strata of the US society and that confidence in the US science system has been weakened during his presidency. At the same time, one should emphasize that the US and Western Europe are different when it comes to the decline of the bottom 50% national income share developments over time – with the US case showing a much more dramatic picture than in Western Europe (see Figs. 3 and 4).

As regards the EU, the largest country with a populist government is Italy, namely as of June 2, 2018. The first Conte coalition was structurally similar to that of the Greek coalition of Prime Minister Tsipras: A combination of a right-wing populist party with a left-wing populist party. It is not surprising that a populist political majority has emerged in Italy since the traditional middle-of-the-road parties have failed to deliver adequate economic policy reforms. In 2016, the per capita disposable real income in Italy was just as high as it was in 1995 so that Italy effectively stands for about two decades of stagnation. It is strange that internal EU/Eurozone pressure for systemic reforms and better policy has been weak in the case of Italy. However, one may also emphasize that the existing framework of policy surveillance, be it by the IMF or by the EU, is looking mainly at short-term and medium-term economic changes. A long run growth perspective is a standard element of policy surveillance nowhere and it was only in the context of G20 – after the Transatlantic Banking crisis – that a broader growth enhancement initiative has occurred, namely at the G20 meeting in Brisbane. While the leading western OECD countries had overcome the shock of the Transatlantic Banking Crisis by 2015 – except for Italy in the field of growth and Greece, the UK and the US (105% in Q2 2018) in the field of the debt-GDP ratio – the prospects for sustained growth seemed to have improved after the many reforms taken in the US and the EU countries after the collapse of Lehman Brothers. However, the Corona shocks in 2020 have created new regional and global challenges.

In the EU, the creation of the European Systemic Risk Board (ESRB) in 2010 was a major institutional innovation designed to look at macroprudential risk analysis and to provide suggestions for adequate policy measures. The US was faster with radical systemic reforms for banking recapitalization and enhanced prudential supervision under President Obama than the EU, the US under President Trump looks as if it would take a step backwards in terms of its economic policy approach: With a broad tax reduction, a first step towards banking deregulation and higher government expenditures, Trump’s policy agenda looked peculiar, but the most sweeping change concerned international trade policy.

President Trump has emphasized in his speeches and tweets how unfair the US merchandise trade balance deficit of some 800 bill. $ per year allegedly is, but all these complaints are ill-placed since the goods and services trade balance is relevant in a “mechanical way” for explaining total job losses through international trade: This mechanical view means that one could calculate – at a given domestic productivity – how many jobs in the services or the manufacturing sector could be created if the net imports of goods and services would have been replaced by domestic production. For example, the study by Lawrence (2017) indicates that the trade balance deficit of the US in this sense corresponds to about 3.5 million US jobs. The economic view of international trade, however, is different to the mechanical view of laymen: With more trade (or more regional integration fostering overall trade; Trade creation in the regional integration club of the respective countries exceeds the trade diversion effects for the outsider countries – a traditional assumption), there is increasing international specialization and productivity growth in each trading partner country so that all countries benefit from trade. In an empirical study for EU countries Jungmittag (2004) has pointed out that particularly high-technology specialization and knowledge diffusion through trade have significantly contributed to the growth of real GDP. A paper by DG2 of the European Commission (Vandenbussche 2014) has indicated that OECD countries’ trade is mainly in technology-intensive goods while trade with China is characterized by the different types of goods traded.

To some extent President Trump seemed to wish the US to jump back to the 1980s – with a higher share of industrial output and the dynamic Reagan years. Several Trump trade advisors and other experts were actually taken from the former Reagan team and one could get the impression that Trump was trying to replace the role of Japan in Reagan’s presidential years with that of China which, however, cannot work:

Japan with its high current account surplus position vis-à-vis the USA was quite dependent on the US in the field of international security policy so that Reagan was able to impose self-restrictions of exports in certain sectors on Japan and also could push Japan to accumulate more dollar reserves. The situation under the Trump Administration with respect to China is, of course, totally different. The trade diversion effect towards ASEAN and EU countries in the case of US import tariffs on Chinese goods will be much larger than similar effects in the 1980s: With Chinese exporters facing high import tariffs in the US, in many sectors many Chinese firms will re-direct large export volumes to ASEAN and the EU countries where prices of products in sectors with relevant Chinese exports will fall. To some extent US import protectionism will stimulate EU countries to also come up with higher import tariffs vis-à-vis China. There will be many reasons for both the USA, the EU and China to use the WTO’s dispute settlement mechanism, but due to the Trump Administration this mechanism no longer works since the end of 2019.

#### New optimum tariff approach

As regards the current account adjustment, standard macro models do not adequately take into account the role of net factor income from abroad and the net effect of profits from subsidiaries in the home country and abroad, respectively. The (cumulated) FDI aspect on the current account has been analyzed by WELFENS (2011, 2017); for a country with net outward FDI stocks, the requirement for a real depreciation to improve the current account is stricter with respect to the goods import elasticities than the Marshall-Lerner condition says.

As regards tariff rates under a No Deal regime, the Johnson government has initially pointed out that the previous May government had already developed a list of import tariffs to be applied post-BREXIT (see appendix). Such a list of tariffs should normally reflect the optimum import tariff literature. A similar observation refers to the Trump Administration where 2019 brought US import tariffs vis-à-vis China which obviously were assumed to reflect optimum import tariffs: As the US is a big economy – certainly more so than the case of the UK – an import tariff reduces the net-of-tariff world market price and therefore could bring a welfare gain for the US.

One may assume that the import tariffs proposed reflect the traditional optimum tariff rates and therefore are determined as t^opt^ = 1/E’ where E’ is the supply elasticity of the foreign big country (here China; or the EU27 or ROW = rest of the world). However, it can be shown that these tariff rates are not optimum tariff rates in a setting with outward foreign direct investment (see appendix). The reason why outward FDI stocks have to be taken into account is simple: If MNCs from country 1 (home country: US) have subsidiaries in country 2 (say, China) which export to country 1, then the overall profits of MNCs from country 1 are reduced by import protectionism in country 1. It can be shown that in the presence of cumulated outward FDI the “true optimum tariff rate” is smaller than 1/E’ (Welfens 2019a; appendix, p. 237; and Welfens 2020a). The adequate formula is.

$$ {\mathrm{t}}^{\mathrm{opt}}=\left(1-\alpha \mathrm{\ss}\ast \right) $$

where ß* is the foreign share of profits in foreign GDP and α is the share of foreign capital (K* abroad) owned by investors from country 1 (home country); for example, if investors from country 1 own 50% of the sectoral capital stock abroad and if ß is equal to 1/3rd, the optimum tariff should be 5/6^ths^ of what the traditional optimum tariff literature suggests. Assuming that the Trump Administration has applied conventional optimum import tariffs to China and that UK’s May import tariff list for the No Deal case is also based on traditional optimum tariff literature, both the US and the UK import tariffs are too high to maximize national economic welfare. One cannot exclude that the Trump Administration has adopted a policy vis-à-vis China which is shaped more by technology concerns – illegal international technology transfers in favor of China – than by traditional trade policy such that reference to optimum tariff rate concepts would be not a major point for choosing US policy measures.

It is also remarkable that the Trump Administration has a tendency to focus on issues of asymmetric trade vulnerability (Diekmann 2020); for example, threats of import tariffs in the automotive sector from countries with a current account surplus, and to combine those issues with the leadership of the US in global financial markets and the globally dominant role of the dollar: Several issues of asymmetric sensitivity are then combined within the US negotiation strategy although no inherent linkages of the various asymmetrical fields exist. As regards the WTO, the US points to the no longer appropriate privileges of many once developing countries which have already have become successful newly industrialized countries and that the WTO rules are inadequate to deal with China’s trade policy which is partly shaped by state-owned firms in key sectors. However, the US position is rather inconsistent when it comes to the WTO dispute settlement mechanism (Diekmann 2020): Between 1995 and 2018, there were 595 dispute cases where the US was the defendant in 124 and the plaintiff in 155 (WTO 2020). Hence 47% of all WTO cases involved the US. In addition, the US had joined 158 cases as a third party. Thus, the US was involved in three-quarters of all cases before the WTO and it is difficult to imagine that the US can rightly complain that the dispute settlement mechanism was biased against the US. The position of the US Trade Representative - USTR (2018) – under the Trump Administration was in stark contrast to the CEA’s position on trade policy in 2015 (Council of Economic Advisers 2015). The CEA emphasized a values-driven trade policy approach which would allow the US to consider environmental protection, non-discrimination and labor standards. However, the USTR emphasized five key fields: Reinforcing national security, reinforcing the US economy, achieving better free trade agreements, aggressive international implementation of US trade law and a reform of the multilateral trading system. President Trump often argued that previous US governments had been incapable of achieving an adequate result at the international negotiation table.

#### Prospects in US trade policy

It should not be excluded that the US Administration post-2020 might want to start free trade negotiations with the EU27 and possibly indeed negotiate both with the EU27 and the UK (obviously only in case that Trump is re-elected). The order of magnitude from a Transatlantic Free Trade agreement could be about 2% of GDP for both the US and the EU (Jungmittag and Welfens 2020). If Trump should not be re-elected, the US political support for EU integration would become visible again and this in turn could undermine the UK negotiation position both vis-à-vis the EU and the US.

The US under the Trump Administration no longer supports the general principles of the WTO, namely non-discrimination, broad reciprocity and the most-favored nation clause plus “single undertaking” which means that a broad approach (broad reciprocity) for making concession and compromises should be adopted and no “micro-management” of trade policy is acceptable. The latter, however, is clearly visible in the US-China Phase-1 Agreement from January 2020 where the US pushes for managed trade and even apparently is willing to rely on large Chinese state-owned firms implementing new Chinese trade legislation which should help to reduce Chinese exports and raise imports from the US. At the same time, the US Congress is threatening that China’s big firms – if they are state-owned – will no longer be listed after a grace period on US stock markets.

As regards BREXIT, this was a historical shock for the EU – and the UK – and it belongs to the class of major politico-economic shocks that have not been understood in broad strata of the political communities and the public in Europe at large. Boris Johnson’s political victory in the December 2019 election erased the option of a second referendum and translated an inconsistent EU referendum of 2016 into political reality on the one hand; on the other hand, BREXIT is a populist project which witnesses the enormous weakening of economic elites in the United Kingdom and moral hazard problems within the Conservative Party which normally supports key arguments of leading British economists. However, the Royal Economic Society (RES) did not want to speak out openly against the result of the referendum; a minor RES initiative concerned complaining vis-à-vis the BBC reporting of BREXIT pros and cons in the Economics community. The credibility of the so-called experts has diminished significantly in the context of the Transatlantic Banking Crisis and in the UK in the context of BREXIT. One may raise the question of why foreign investors should consider the UK to be a preferred investment location if basic economic arguments can be very broadly ignored by a government under a prime minister from the Conservative Party. One could also raise doubts about the political risk management in Brussels by the European Commission in a broader perspective, since the EU did not really monitor the run-up to the EU referendum and few if any of the leading EU politicians seem to have anticipated BREXIT; worse, the European Commission has refused to draw key lessons from BREXIT and thus there might be continuous instability of the EU integration club. This holds all the more since the bad performance in Italy, Spain and France during the corona pandemic has undermined broad political support in these countries for the respective governments which seemed to be particularly weak – as coalition governments – in Italy and Spain. For small EU countries, BREXIT raises the unpleasant prospect that they might have to face a strong Franco-German twin EU policy approach (with the UK no longer available as a political hedge option). The more isolated the UK outside the EU will be, the more British governments might try to undermine the EU in order to have a minimum number of new EFTA countries for a new regional integration project – an FTA – in Europe. It is clear that the UK government wants a fast and broad FTA with the US, but the United States is not likely to make broad concessions quickly – and also one has to take into account that the UK is a politically rather divided country since the EU referendum. At the same time, from a US perspective, the UK is no longer in a position to indirectly represent key US fields of interest at the EU negotiation table in Brussels.

## BREXIT problems

The UK held a referendum on the question of continued EU membership on June 23, 2016, where a 51.9% majority was in favor of leaving the EU (i.e. BREXIT). A closer look at the run-up to the referendum and the BREXIT dynamics reveals several crucial insights which raise serious doubts about the legitimacy of BREXIT, the conjecture that the UK was suffering from EU immigration and that “Global Britain” would be a convincing strategy for a post-BREXIT United Kingdom (the UK post-BREXIT might indeed face the risk of a new Scottish referendum and could disintegrate if there would be a majority for Scottish independence – Scotland had a clear pro-Remain majority in the EU referendum of 2016). By mid-June 2018 – four months ahead of the critical October deadline for an agreement - the British government has been unable to come up with a clear proposal on certain issues in the withdrawal agreement. The UK is facing a dangerous slow-down of the BREXIT process which could make financial markets more volatile; this volatility could have a destabilizing overlap with negative stock market dynamics facing enhanced negative impulses from an aggressive US trade policy. The following analysis is largely based on Welfens (2017a; 2019).

The political basis of BREXIT is very weak since the 2016 referendum was disorderly. For reasons which remain unclear, Prime Minister Cameron suppressed the findings of the Treasury Study of April 2016: BREXIT = 10% income loss; not a single word on the key findings appeared in the 16-page government information brochure for voters prior to the referendum. The normal result – based on UK popularity functions/10% info - would have been 52.1% for Remain on June 23. There has been an intense debate over EU immigration which Mr. Cameron portrayed as being a major burden for the UK. However, the OECD has shown that immigration to the UK actually brings net benefits for the British budget. This has not prevented Mrs. Theresa May – who had been the Home Secretary (interior minister) in the Cameron governments for six years – from repeating the claim about the massive long-run EU immigration burden in the White Paper of 2017 which, however, also shows a graph according to which non-EU immigration had been the dominant phenomenon. The anti-immigration rhetoric of Cameron has mainly served to create a scapegoat for the massive cuts in government transfers to local communities after the Banking Crisis: −3.5 percentage points of national income which resulted in an under-provision of local public goods; and this problem was then blamed on EU immigrants. The strong infighting within the UK government is one problem; another is that the Global Britain strategy emphasized by the May government looks quite unconvincing and unrealistic as President Trump is undermining the World Trade Organization whose dispute settlement procedure will become ineffective from 2019 as the US has blocked the election of new judges to the appellate body. Without a functioning WTO, the Global Britain strategy is bound to fail as the UK is too small to solve potential future trade conflicts on the basis of bilateralism. The UK accounts for less than 2.5% of world gross domestic product (GDP) at purchasing power parity. There is considerable risk that the UK will effectively leave the EU single market in 2021 under a No-Deal scenario which would impose very high costs on the UK: 16% to 25% of real income losses over the long run (see Welfens (2017b) and Erken et al. (2017)).

As Boris Johnson took power from Prime Minister May in summer 2019, and then obtained a strong victory in a general election in December 2019, the UK seemed for some time be in a strong position vis-à-vis the EU27. However, the weak performance of the UK in the corona pandemic in the first half year of 2020 – with about 40,000 COVID-19 deaths by early June 2020 (high compared to 9000 COVID-19 deaths in Germany; and even higher than the very high figures in Italy) – has undermined the Johnson government’s popularity in the UK and it remains to be seen whether or not the UK government, relying on an expansionary policy mix supported by the Bank of England, will be able to achieve a quick economic recovery in 2021. The larger the economic problems in the UK will be, the more radical could be Johnson’s economic agenda in terms of the deregulation of financial markets and labor markets, respectively. Such policy moves would create considerable political conflicts with the EU27.

With BREXIT, the EU will lose 1/5th of the community’s national income so that it will no longer be the world’s largest single market – instead, this will be the US where President Trump seems determined to destroy the post-1944 multilateral system and to replace this with a new system of great power rule; with the US being supported by ‘vassal countries’ from Latin America, Europe, Asia, Africa and elsewhere. The UK could face a situation where it would also become the vassal of the US (“reverse colonialism”). Financial instability could be a major international problem – BREXIT uncertainty plus destabilizing impulses from the US – via an aggressive trade policy on the part of the Trump Administration – contributes to financial market instability. An EIIW research paper shows by employing an event methodology (Korus and Celebi 2018) that “hard BREXIT news” could have a stronger impact on the British Pound than more favorable “soft BREXIT news”. Moreover, the empirical paper from Welfens and Baier (2018) show the impact of BREXIT on inward foreign direct investment inflows. OECD figures for 2017 are noteworthy: UK FDI inflows −92%, compared to 2016 (global FDI inflows: −18%). The decline in 2017 obviously reflects the effect of ongoing uncertainty about future UK access to the EU27 market, while the increase in FDI inflows to the UK is largely explained by the strong Pound depreciation – the positive link between Pound depreciation and FDI inflows is reflecting the Froot and Stein (1991) arguments for a setting with imperfect capital markets.

### BREXIT problems: Policy consistency aspects and new challenges

The UK left the EU on January 31, 2020, and thus a historical decision has been taken by the UK government. This decision can hardly be explained by any rational economic analysis, only a political analysis seems to explain this move; moreover, strange elements in the referendum 2016 have played a key role – and the determination of Boris Johnson as a political frontrunner for BREXIT and as a big winner of the UK election in late 2019 - which went along with a political purging of the Conservative Party. One may still argue that the referendum had major political information pitfalls and the three year-long political stalemate after June 2016 reflects some of these problems.

The stalemate years could have been expected as in normal circumstances in the EU referendum of 2016 would have been 52.1% pro-Remain, but Prime Minister Cameron did not include a single word on the findings of the Treasury study which were available in early April 2016 - an income loss of 10% was the key finding – in the crucial 16-page government information brochure for voters. This policy of not providing such critical information to the electorate stands much in contrast to the Scottish Independence referendum of 2014 where the government’s information brochure warned of a £1400 GBP per capita income loss and “the loss of all the benefits of EU membership”. The higher losses projected by the Treasury analysis in the case of BREXIT were suppressed in the brochure of 2016.

Remarkable was the position of PM May’s Defense Secretary Gavin Williamson as he had publicly announced that the UK’s aircraft carrier HMS Queen Elizabeth will sail in its maiden voyage not only to the Mediterranean but also to the Pacific in order to defend the UK’s position as a global power; Williamson referred explicitly to BREXIT in his speech in February 2019. Post-BREXIT, the UK is only about 1/5th of the economic weight of the EU28 so that the country’s international position at the diplomatic table is clearly weakened.

With BREXIT the British Pound’s international role will be weakened (Eichengreen 2019). The Eurozone and the US stand to benefit from the anticipated fall of the British Pound’s global market share in the global reserve currency market. A rise of the UK government bond interest rate of 0.3% would be equivalent to an additional burden for government that equals the UK’s net contribution to the EU. Implied financial volatilities in the UK, the Eurozone and the US in December 2018 were as high as in the first quarter of 2008. Thus, 2019 could become another year marked by high volatility. The European Systemic Risk Board (ESRB), created in 2010 in the wake of the Transatlantic Banking Crisis as part of the institutional lessons learned, seems not to have worked well in 2018 so that its mandate of macroprudential supervision – analyzing systemic risk in the EU28 – was not really fulfilled.

With BREXIT, UK institutions are no longer be part of the ESRB and this reinforces the problems of macroprudential supervision from an EU27 perspective since more than 60% of wholesale banking markets are still based in the UK, and the City of London in particular. Financial market problems in the UK related to BREXIT would thus certainly spillover into investment and trade dynamics of companies in the EU27 area and this in turn would have negative feedback effects on the UK and the US.

Germany, as a leading EU economy, is facing an economic slowdown in the context of the Sino-US trade conflicts and anticipated BREXIT problems as well as the expected negative spillover effects in the Netherlands and Belgium (plus Ireland) which together account for even higher exports of German firms. All EU27 countries plus the UK are facing the Corona shocks in 2020 which makes adjustment more difficult for most European countries. For the Johnson government, the corona shocks give a historical opportunity to push for a no-deal BREXIT in 2020/21 since the main adjustment shocks of BREXIT would overlap with corona-related adjustment shocks such that voters will have an unclear picture how economically bad for the UK the BREXIT decision was. The inconsistencies of the UK referendum of 2016 and Cameron’s referendum information policy, as well as the economic pain of disintegration, will soon be forgotten by the voters, so the argument goes in part of the UK government Tables 4 and 5.

### An accidental BREXIT

In the UK, 2016 brought – mainly due to Prime Minister Cameron’s information pitfall in the EU referendum campaign – a narrow majority for BREXIT (i.e. that the UK should leave the EU28) which is a populist political project strongly guided by wishful thinking on the side of Brexiteers. Had Cameron followed the information standard of the Scottish independence referendum – here the Cameron government explained that every Scot would lose GBP 1400 and all the benefits of UK’s EU membership in the case of Scottish independence – the result would have been 52.1% for Remain in the EU referendum on June 23, 2016 (Welfens 2017a). The reality was 51.9% in favor of BREXIT and the successor of Prime Minister Cameron is Mrs. Theresa May who has declared that ‘Brexit means Brexit, and we will make a success of it’. The BREXIT negotiations were rather difficult and confusing over the first 24 months, but the new Johnson government brought an implementation of BREXIT on the basis of a new EU-UK Withdrawal Agreement on January 31, 2020; with a one year transition – as emphasized by the Johnson government – so that in 2021 the EU-UK free trade agreement would hold or, in the case of no agreement on the future relationship, a No Deal situation would characterize EU27-UK economic relations; until such time as a free trade agreement would be achieved. The EU mandate (European Council 2020) emphasizes that the European Union has an interest in maintaining a high level of regulation in both the EU and the UK (“level playing field”) as well as the EU’s interest in access to UK fishing territory; the UK (HM Government 2020) in turn has emphasized that the UK will not accept jurisdiction of the European Court of Justice and that the UK expects a broad Free Trade Agreement which should go beyond the CETA Treaty between the EU and Canada.

The European Union argues that the UK cannot get generous market access to the EU single market unless the UK signs an agreement on regulatory policy cooperation and key principles of tax policy orientation so that a tax race to the bottom could be avoided. The EU obviously has no interest in a fast negotiation, namely to the extent that the US presidential election on November 3 plays a key role in terms of the negotiation position of the UK. If Trump is not re-elected as US president, the position of Prime Minister Johnson in the negotiations with the EU will be rather weak. One may argue that this is the logic behind Johnson emphasizing in early March 2020 that his government expects fast progress in the negotiations by June 2020 – otherwise the UK would prepare for a No Deal approach to be implemented at the end of 2021; and obviously the UK would hope that a US-UK free trade agreement could be achieved by then, too.

While UK-US free trade negotiations will start in spring 2020, it seems rather implausible that a quick agreement will be possible, not least since the UK has a bilateral trade surplus vis-à-vis the US; and President Trump has an interest in reducing this bilateral trade deficit. Whether or not any synchronization of EU-UK FTA negotiations and UK-US-FTA plus EU-US FTA negotiations could be implemented remains to be seen – from a consistency perspective this would be desirable for the US, the UK and the EU and could also have favorable results (Ries et al. 2017), but the US election calendar makes this rather unlikely.

Key statistical data on UK-EU and UK-US trade present a clear perspective for the UK: It is obvious that with 45% of the UK exports going to the EU27 (see appendix) there should be a strong interest of the UK to negotiate a broad free trade agreement with the EU, at the same time, the Trump Administration will exert pressure on the UK to further open up the British market. British economic interests in turn might come at the cost of EU countries in certain fields; for example, the double UK-Dutch headquarter issue of Unilever was decided in favor of London after BREXIT. As the UK is facing the biggest recession in about 300 years in 2020 (Bank of England 2020), the British government will push for more free trade deals in a rather short period of time since more deals raise hopes for cheaper imports and higher exports which could both reinforce economic gains for the UK and a long run upswing, respectively. As regards the EU, it holds that the whole field of trade negotiations could become more complex through the supply-side and demand-shocks related to the coronavirus in 2020 which is expected to strongly slow down growth and employment in OECD and ASEAN countries as well as China.

Once the UK has left the EU completely in 2021, the United Kingdom could be a rather isolated country in Europe and one may expect that under a Conservative government the UK will push strongly to expand its influence in Asia and ASEAN, respectively, where the UK would become a strong rival of the European Union. The Johnson government has announced that it will reduce immigration from the EU and particularly the immigration of unskilled workers, rather the UK government would like to invite immigrants from many other countries; ideally skilled workers (here, Hong Kong is a natural focus of the Johnson government).

The EU, in turn, would also face the challenge of the UK lowering corporate tax rates and undertaking a new wave of banking deregulation which will put significant adjustment pressure on the EU27; provided that the US follows a similar policy approach under President Trump and his successors. President Trump has already started US banking deregulation in 2019 and the corona crisis has made most OECD countries more willing to weaken banking regulation as part of a broader approach to restart the economy and to overcome a very serious economic crisis. The UK economy’s export interests would be massively guided towards higher exports to Asia and indeed in early 2019 the May government has, for example, pushed Malaysia – and other former British colonies in Asia –to quickly accept free trade agreements with the UK post-BREXIT; with no immediate result. As a new EU-UK rivalry in ASEAN will undermine EU-ASEAN cooperation and the ability of the two regional country groups to keep China away from the option of switching from multilateralism to bilateralism; China’s government has emphasized its support for multilateralism in 2017/18, but a US approach bilateralism under Trump generates incentives for China to also adopt a similar approach to bilateralism which, however, is certainly not in the interest of the EU or ASEAN. If the US and China both switch to bilateralism, this would lead the world economy back into the nineteenth century – that is moving to a Great Powers regime, namely with the leading powers being the US, China, Russia, possibly also Brazil plus Japan (unlikely to be a stable international system). President Trump’s May 2020 proposal to postpone the G7 meeting and also to invite Russia, the Republic of Korea and India shows the populist President’s willingness to bring Russia back into international political networking – despite the changing of Russia’s border in the context of the annexation of the Crimean Peninsula. By not sticking to Western political principles, Trump undermines the credibility of the US and the Western international policy. At the same time, it is doubtful that Germany and other European countries will want to support this Trumpian political initiative.

As regards British universities, in 2018 the May government already massively increased attention towards students from Asia who can expect a friendly welcome at many British universities which so far have also looked for students from the EU and who, in Economics, would so far present themselves to be a natural intellectual bridge to the European Union which post-BREXIT will no longer be very convincing.

However, the corona crisis has brought massive initiatives in global online teaching and here major US universities and British universities could both conquer international higher education markets while state-organized universities in the EU27 countries largely are hesitant to enter the new digital global education market. This new internet-based teaching and research market is not just about higher education, it will also be about exporting political values and creating new international networks. Here, the EU27 countries seem to be in a rather weak position in the post-corona world. If the EU27 countries should be concerned for many years with internal conflicts in the EU27 and the Eurozone, respectively, it is quite likely that the US, the UK and other countries will have achieved global higher education digital leadership by 2025.

That the UK’s orientation towards Asia will have a military twist is also hard to overlook. The UK will further raise its military budget in order to offer military protection to certain countries, such as in Asia or Africa, namely as a means to then get more favorable conditions for new free trade agreements to be realized post-BREXIT (with a low economic weight of the UK compared to the previous EU28 joint power position; the UK alone is 1/5th of the EU28). Such a new UK-Asian outreach approach in turn could destabilize the ASEAN group as the UK has strong traditional historical links to some of the member countries only and Asia itself could become destabilized by a higher Western military presence from the UK side whose only half-baked logic is somewhat linked to a rather strange BREXIT. If British ships can sail somewhat provocatively in the Pacific, Chinese ships could one day sail in the Mediterranean. BREXIT thus undermines multilateralism in opaque and risky ways, along with the more open anti-multilateralism of the Trump Administration – all this bringing a massive shock to global governance.

BREXIT came as a rather surprise for the EU and also for the Cameron government itself, as well as most capital market participants who obviously did not understand the broad mood of dissatisfaction of British voters with the EU. The EU indeed faces much broader dissatisfaction since several Eastern European countries have refused to accept any burden sharing with regard to the refugee wave of 2015 and thereafter. The majority decision of the European Council taken in 2016 for an assignment of refugees to all EU countries has not been enforced, instead the German Chancellor Angela Merkel – who in 2015 opened the German borders for about a million refugees coming from Hungary and other EU member countries – in the June EU summit in 2018 sought support for a new refugee allocation agreement for which about half of EU member countries signaled a willingness to agree.

With the US undermining the World Trade Organization (see appendix) and imposing import tariffs on Western allied countries, the traditional Western world with a shared support for economic globalization and for more long-run liberalization of trade is no longer a strong political group. There are several questions to be raised in the context of the election of Donald Trump as president and of BREXIT as a historical step of the UK leaving the EU of which it had been a full member for more than 45 years (it joined in 1973) – and looking also at the broader EU-internal conflicts that make the European Union look rather instable; and one could not even rule out that the EU could disintegrate entirely in the medium term.

The US current account balance deficit will increase in the medium term – as indicated by the IMF’s Article IV mission report of 2018 (IMF 2018); one may add that the merchandise trade balance deficit of the US, greatly emphasized by US President Trump, will also increase further. The figures for 2018 - published in March 2019 - indicate a deterioration of the US trade balance which is not surprising in view of the expansionary fiscal policy adopted in 2018. This is likely to make the Trump Administration’s trade policy even more protectionist vis-à-vis the EU/Eurozone and Germany, respectively. To put the focus on the current account from a US-Eurozone perspective would be appropriate for a transatlantic policy dialogue (the US-EU CA surplus for the EU is irrelevant): At least the exchange rate (€/$) could in principle play a role for adjustment; however, the US needs a real $ depreciation, while rising US interest rates and transitorily higher US growth will bring about an appreciation. US protectionism will reduce the export-GDP ratio and the import-GDP ratio in the US as well as in other countries and could also raise the volatility of equity markets and financial markets worldwide – through the dominance of the US as a financial market place; such destabilization is certainly unwelcome in a period where BREXIT will also undermine global financial market stability. With BREXIT, the EU wholesale financial market – which is largely based in the City of London - faces particular problems as post BREXIT, the EU27 faces the strange situation of €-denominated derivatives, currency transactions etc. being located and regulated outside of the European Union, a situation which itself could raise new uncertainties and could contribute to conflicts. The more the corona crisis undermines economic stability in the UK and its banking system, the more the EU27 countries should be worried about having more than 50% of the Euro wholesale financial market based in the UK.

## Coronavirus pandemic, global Corona recession perspectives and EU crisis

### Pandemic problems and EU fiscal policy response

The coronavirus pandemic which hit China in the first quarter of 2020 and other countries, including the EU and US plus the UK in the first half of that year, has brought a Corona World Recession which means that output in most IMF member countries will decline in 2020; the output reduction in the EU, the UK and the US is expected to be stronger than the Transatlantic Banking Crisis (IMF 2020a; European Commission 2020). As regards the EU, it was interesting to observe that the initial anti-epidemic policy of EU countries was rather uncoordinated and that both France and Germany imposed restrictions on the export of medical equipment and protective supplies for those working in the hospital sector in March 2020 so that the hard-hit Italy, for example, could not get desired imports from its EU partners. As in every recession, protectionist pressure is mounting such that one has to anticipate that the corona economic shock which comes parallel to the medical epidemic shock will undermine global trade growth. Moreover, one may expect that the overlap of the corona epidemic shock – associated with lockdowns and shutdowns for millions of firms and households in OECD countries and China plus other countries – and the Corona World Recession undermines existing international value-added chains so that new international delivery risks have become obvious that could stimulate firms to rely less on the import of intermediate products from abroad which would reduce global trade; some firms, however, might want to look rather for more international diversification in the sense that mainly relying on a few suppliers, for example from China, seems to be too risky a strategy for international value-added chains. At the same time, the Trump Administration’s trade conflict and the additional political conflicts over the outbreak of the epidemic in China undermines trade and investment links between China and the US in particular. New US legislation from 2020 intends to limit the US stock market listings of Chinese firms which could reinforce the interest of China in trade and investment relations with ASEAN and the EU in the long run. With an apparent economic and political weakening of the US in the corona crisis in 2020 – and indeed no US leadership in the international economic crisis, the first such scenario since 1945 – both the EU and ASEAN are facing serious problems as the lack of US leadership requires that EU countries and ASEAN countries would both act in a more coordinated and consistent way.

This, however, is not yet visible in the EU where enormous fiscal policy packages have been adopted or are considered by the European Commission while the risk of a new Euro Crisis 2 is not adequately taken into account: The EU countries have adopted a €540 billion loan package in April 2020 (€100 billion as loans for EU unemployment insurance schemes in heavily hit EU countries, €240 billion as loans from the European Stability Mechanism – the ESM – which gives loans for corona-related health problems which is a strange and inadequate conditionality) of which €200 billion are declared to come from the European Investment Bank (EIB) where the EIB itself gives €25 billion and argues that the remaining €175 billion will come in the form of complementary loans from private banks for small and medium-sized firms. However, there is a substitution effect as most of these private bank loans would have been given anyway to such firms so that the effective EIB impact is rather close to about €60 billion so that the effective EU loan package is not €540 billion but rather €400 billion; compared to the EU headline loan package, thus almost 1% of the EU’s GDP thus is missing. In May 2020, the European Commission suggested that the EU should come up with a €750 billion recovery fund which would give 2/3rd^s^ of this amount as grants mainly to Italy, Spain, France and Poland; 1/3^rd^ would be in the form of additional loans which taken altogether is a strange package. Poland and Hungary obviously get large transfers of €38 and €8 billion, respectively, although neither country was particularly negatively affected by the epidemic nor by the corona recession (if one follows European Commission Spring 2020 projections: European Commission 2020). Almost €50 billion of transfers and loans are going to Eastern European EU countries simply to secure the political support of these countries; as the Commission gives large sums to Poland and Hungary, as populist countries which undermine EU principles of the rule of law and democracy, respectively, the European Commission thus rewards anti-EU political behavior within the European Union. It is not easy to understand why the European Commission has allocated a rather high share of funding – expressed in recipient countries’ GDP – to Eastern European EU countries. However, the post-BREXIT Banzhaf power index ranking could be a simple explanation here: According to Kirsch (2016), the implementation of BREXIT means that Germany and France will gain in terms of power as measured by the Banzhaf index, which refers to the countries’ political weight under weighted majority voting in integration clubs and international organizations, respectively; however, the relatively highest power increase in this context is for Spain and Poland.

Eastern European EU countries can get additional funding post-BREXIT as non-Eurozone countries so that these countries have an incentive not to even join the Eurozone in the case that standard optimum currency area literature would suggest that Euro membership would be adequate. As long as the extra-transfers obtained as a non-Eurozone member country are higher than the benefit to be expected from Eurozone membership, countries will stay outside the Eurozone: This creates a dynamic membership consistency problem which could only be solved if the Eurozone countries created their own Eurozone Parliament and a kind of Eurozone government so that the Eurozone countries gain a new outside option in the EU: They could threaten to leave the EU and recreate the Union as an economic and political Eurozone integration club.

The EU’s Quest macro model considers effects of the lockdown/shutdown in the epidemic and key aspects of the pandemic shock, respectively (Pfeiffer/Roeger/In’T Veld, 2020): The effect of several policy options are considered, namely short-term work allowances plus liquidity guarantees (as well as some other policy elements). Such interventions reduce output losses from the Pandemic shock by about ¼. Following the logic of this model one can anticipate that the EU’s € 750 bill. loan package – of which € 360 bill. will be transfers, according to the EU summit agreement in July 2000 – could help to stabilize output in the EU, particularly to the extent that EU countries will adopt projects in favor of higher innovation and growth dynamics. It is, however, not very likely that this will be the case since the EU Commission’s main emphasis has been on digital modernization, climate protection and modernization of health systems while no clear priorities are in favor of innovation and growth policies. Concerning the depth of the recession in EU countries in mid-2020 the Eurozone is the main area of concern while the EU Commission allocates high transfers – relative to the recipient countries’ GDP – to eastern European countries; Italy and Spain can expect about 4% and 6% of GDP, respectively. Such transfers alleviate budget problems of these countries in the short run, but the anticipated debt-GDP ratio of about 155% for Italy at the end of 2020 – up from 135% in 2019 – will raise the probability of losing investor grade rating for sovereign bonds (a similar shock could come from the anticipated massive increase of the US debt-GDP ratio by the end of 2021 which could bring about not only a loss of the top rating for the US but also a broader downgrading of sovereign debt in several Eurozone countries). As soon as Italy loses investor grade rating there is a serious risk that Italy will lose access to international capital markets which in turn would trigger a new Euro Crisis. With an alternative JEBs approach coupled with emphasis on priority additional public investment spending on Trans-European Networks (electricity, digital, highways, trains) the potential problem of a new Euro Crisis could have been avoided.

It is noteworthy that the €540 + 750 billion of EU funding suggested in Brussels in the first half of 2020 does not really address the biggest risk of the corona recession, namely a new Euro Crisis – with Italy in the epicenter; and one should note that the €1290 billion proposed EU loan packages altogether exceed the overall IMF loan capacity of €910 billion ($1000 billion). If Italy should lose investor grade rating (at Moody’s and Fitch it was already just above investor grade rating as of April 28, 2020 when Fitch made a downgrading for Italy’s debt to BBB- which is just above non-investor grade; only S&P in the group of the three leading rating agencies had Italy two notches above investor grade at that time) the country could quickly lose access to international capital markets. It would need annual refinancing of about €350 billion and with respect to the current government deficit of about €100 billion – say in 2021 and 2022 - which would already be beyond the remaining lending capacity of the ESM so that Germany, France and other EU countries would have to increase equity capital by about 200% of what it is now; for Germany this would mean an additional €48 billion. The €1290 billion would represent 7.8% of the EU27’s national income in 2019 which in 2025 itself would bring the EU/Eurozone countries further above the critical 60% deficit-GDP ratio of the Maastricht Treaty which is the basis of the Eurozone and the European Central Bank (Germany’s national deficit-GDP ratio will be close to 11% of GDP in 2020 which could encourage other EU countries to go even further – e.g., Italy, Spain and France – so that the Germany’s fiscal policy seems to be doubtful and too extensive when considered in relation to the somewhat comparable recession shock of 2009). On top of this comes the debt-GDP ratio existing in Eurozone countries and non-Eurozone EU countries in 2020 which is a major EU contradiction:

- The European Commission and the EU, respectively, create serious obstacles for the seven non-Eurozone EU countries which are expected to join the Eurozone in the medium term, namely once they meet the so-called convergence criteria for Euro candidate countries (a deficit-GDP ratio not exceeding 3%, a debt-GDP ratio not exceeding 60% and an inflation rate not more than 1.5 points above the three Eurozone countries with the lowest inflation rates; plus a long run interest rate not more than 2 points above the three Eurozone countries with the lowest inflation rates; the independence of national central bank and no devaluation of the currency vis-à-vis the Euro for two years; note that Denmark has an opt-out clause). Both Sweden and Poland have met the convergence criteria already, but were politically not ready to join the Eurozone. This creates a serious problem since on the one hand the Eurozone cannot achieve an optimum policy mix of monetary and fiscal policy – the Commission’s €750 billion EU loan approach, as suggested in May 2020, forces the Eurozone countries to combine the ECB’s monetary policy with an implicit fiscal policy program of 27 EU member countries, while only a joint fiscal policy of EU19 countries in the Eurozone is required. There is no Eurozone Parliament and no Eurozone government which could assume a relevant and necessary role for fiscal union in the Eurozone. The initial idea of the Economic and Monetary Union which had started in 1999, namely that all EU countries rather would soon be Euro countries, has turned out to be an illusion. It creates a serious internal contradiction that the European Commission and the European Council/EU Council of the Ministers of Finance, which suggested the €540 billion EU loan package in April 2020, for many years indirectly undermine the prospects of non-Euro member countries to join the Eurozone (one may argue that the EU’s €540 billion package is only a €365 billion package as the European Investment Bank gives only €25 billion in the EIB’s overall €200 billion loan package – with €175 billion coming from private banks; in addition to the €200 billion EIB package, there are €100 billion as EU reinsurance of national insurance systems plus €240 billion as loans from the European Stability Mechanism; €750 billion plus €540 billion as EU loans thus amounts to about 7% of the EU’s GDP and therefore also raises the debt-GDP ratios of EU countries by at least 7 percentage points).
- It would have been much better for the European Commission to come up with a small EU loan package of corona-related Euro transfers for Italy, Spain and Greece as countries heavily hit be the corona shock; and to avoid a Euro Crisis 2 through the introduction of a “Joint Euro Bonds”, based on 55% collateral through national gold and currency reserves of Eurozone member countries and the creation of a JEBs Fund outside the EU, namely through the 19 Eurozone countries (Welfens 2020b); the European Central Bank could buy up to 40% the JEBs. This would have been adequate for dealing with the Euro Crisis 2 risk provided that Italy and Spain would have obtained a relatively high share of the JEBs proceeds, namely on the basis of certain growth-policy related conditionalities (Germany and the Netherlands would have received a share below their respective shares in the Eurozone GDP). JEBs could thus have mobilized about 5% of the Eurozone’s GDP over four years – altogether about €600 billion and this would have brought Italy a much-reduced interest payment burden as a quasi-transfer in the EU. One could adopt an €80 billion additional EU loan – instead of the €750 billion suggested by the Commission – of which three-quarters would go as transfers to Italy, Spain, France and Belgium plus Greece. The net gain would be an EU loan which is €70 billion lower than the €1290 billion proposed EU loan packages; and on top a Euro Crisis 2 would be prevented by anchoring the Eurozone interest for several years at a very low level, comparable to that of the UK and the US; the policy mix would also be roughly the same as in those two countries. Effectively, this would also be a hedge against the potential US loss of its AAA rating which would trigger broader downward moves in sovereign ratings in other OECD countries, potentially including Eurozone countries (above all Italy). A small increase of the seven-year EU budget from 1.0% to 1.1 or 1.2% of the EU’s GDP could be additionally considered and this in turn would give room to support Italy and Spain, but also to help Eastern European EU countries through higher structural funding for their modernization approach. JEBs would bring considerable reductions of interest payments for Italy and Spain and a few other countries and thus would be part of implicit transfers for these countries hit rather heavily by the corona crisis. From the proceeds from JEBs placed in the market – with maturities of 2, 10 and 30 years as a strategy to develop Euro bonds markets worldwide and reinforce the Euro’s attractiveness as a reserve currency, both countries could obtain a higher share than would correspond to their respective share in the Eurozone’s GDP, namely if both countries would provide additional (progressive) collateral coverage; for example, on the basis of modest multi-year wealth taxation. In Spain and Italy, the ratio of net wealth of the private sector to disposable gross income in 2019 was 10 and 8, respectively (European Central Bank 2020). The JEBs approach is cheaper than the €750 billion loan package of the European Commission; moreover, it helps to avoid a Euro Crisis 2 and also contributes to overcoming the corona economic crisis in the EU relatively quickly which, in turn, will help to bring down the debt-GDP ratios of Eurozone and EU countries.

The whole situation points to the problem of the lack of a Eurozone Parliament which indeed should be considered as a rational element of future fiscal federalism within broader EU reforms. Without such a Eurozone Parliament and an accountable Euro government, the EU is likely to disintegrate, at the latest in the decade after 2025 when demographic divergence will increasingly characterize the EU– with rapid ageing in Germany, Spain, Italy and Greece, but much slower greying of society in France so that political consensus will become more difficult to achieve in the long run.

It is noteworthy that a survey of the European Parliament (2020) shows that only 33% of the respondents in EU countries covered by the public opinion survey in early April 2020 consider direct EU economic support for EU member countries as a top EU priority; even in Italy and Spain only 49% and 43%, respectively are see direct economic support as a top priority (with up to three items that could be marked; No. 1 turned out to be securing an adequate supply of medical equipment in the corona crisis and No. 2 was making sure that all people in all EU countries will have equal access to a vaccination). The European Commission thus has presented a €750 billion loan package to fund supranational support for EU member countries which is economically doubtful and not very efficient; and which only a minority of the population in EU countries considers as important.

As regards the EU and the Eurozone, respectively, there is also a legal conflict over the ECB’s monetary policy and thus indirectly about the overall construction of the EU: The EU is not a state, but its sovereignty is derived from the member states and the EU’s quasi-constitution, the Treaty of Lisbon; and as the EU is not a sovereign state in its ow right, national constitutional courts might have a say in certain issues. The German Constitutional Court raised doubts about the ECB’s quantitative easing (QE) programs in May 2020 so that there is a constitutional conflict: The European Court of Justice had previously given green light for such QE programs, the German Constitutional Court in turn has argued that the ECB has not considered the principle of proportionality in its QE policy – read: It has not explained carefully why certain QE measures were necessary (the ECB usually points to the problem that the inflation rate is too far below the target ratio of below, but close to 2%) – and also not taken into account the side effect of QE policy on other goals of economic policy of Eurozone member countries. The German Constitutional Court’s ruling could force the Deutsche Bundesbank to no longer participate in the ECB’s quantitative easing programs, including the new Pandemic QE package which consists of a planned amount of €750 billion plus another €600 billion (with a time horizon until mid-2021); the two packages together seem to be represent a very large package, actually too large since the ECB might face a situation in which it would buy too many government bonds, namely almost all new bonds issued in the government bonds market in the Eurozone so that markets no longer determine interest rates and the issue of monetary financing could come on the political and legal agenda. Given the extraordinary corona shock, the ECB is buying both government bonds and investor grade corporate bonds – where the relevant rating status is considered to be pre-corona shock rating. This might be adequate for about a year or so but such artificial rating benchmark should not be used beyond 2021 since otherwise the ECB could accumulate many assets with high potential and actual value losses in its balance sheets.

The international help for countries facing the corona pandemic was certainly not well organized – for example, amongst the group of industrialized countries. It is remarkable that NATO/NATO+ has played some role in the first quarter 2020: While the EU has not been very visible in the early stage of the coronavirus pandemic, other institutions of the West have been more responsive; this includes the work of the Euro-Atlantic Disaster Response Coordination Center (EADRCC) which represents the 30 NATO countries plus 20 former socialist countries, including Russia. In 1998, that institution had been created by the Euro-Atlantic Partnership Council (EAPC) as part of its international policy on “Enhanced Practical Cooperation in the Field of International Disaster Relief”. The NATO website gives the following summary for NATO + (NATO 2020):

- *“Allied armed forces are playing a vital role in supporting national civilian responses across the Alliance. This support includes logistics and planning, field hospitals, the transport of patients, disinfection of public areas, and at border crossings.*
- ***NATO’s Euro-Atlantic Disaster Response Coordination Centre (EADRCC)*** *is an important tool helping to provide relief to Allies. It is NATO’s principal civil emergency response mechanism in the Euro-Atlantic area. The Centre operates on a 24/7 basis, coordinating requests from NATO Allies and partners for help, as well as offers of assistance to cope with the consequences of major crises such as the COVID-19 pandemic. For example, in response to Spain and Italy’s requests for assistance made through the EADRCC, the Czech Republic bilaterally provided both countries with medical supplies, including 10,000 protective medical suits each. Through the same mechanism, Turkey is providing Italy and Spain with medical supplies such as masks, personal protection equipment and disinfectants.*
- *The* ***NATO Support and Procurement Agency (NSPA)*** *has a leading role in responding to the crisis. The NSPA provides logistics support and the organisation of transport of key supplies and equipment to Allies, partners and other international organisations. For example, the NSPA has helped Luxembourg increase its hospital capacity by providing field hospital tents, mobilising equipment in less than 24 h.*
- *NATO supports the* ***Strategic Airlift International Solution (SALIS) programme****. The programme enables participating Allies to charter commercial transport aircraft.* ***For*** *example, the Czech Republic and Slovakia have used SALIS to import planeloads of medical supplies, including facemasks, surgical gloves and protective suits.”*

### The political scars from SARS and the coronavirus pandemic

Another pandemic problem is related to the fact that epidemic shocks have a long-lasting impact on the younger generations of voters who are at an “impressionable age”: These groups typically register an epidemic (e.g., SARS in 2003; coronavirus in 2020) shock with a persistent effect with respect to weakening trust in political institutions which lasts about two decades and which is found to be stronger in countries with weak government than in countries with a strong and effective government in the period of the epidemic shock (Aksoy et al. 2020) while autocratic countries are found to not display much of this type of political shock effect from epidemics and pandemics, respectively.

In OECD countries, the key drivers of COVID-19 fatality rates (ratio of COVID-19 deaths to population) were obesity, the share of the population aged 65 and over and air quality problems (Bretschger et al. 2020); countries with herd immunity policies (UK, the Netherlands, Sweden) had higher fatality rates than other countries. As regards the implications of the above empirical political scar findings one may point out three key aspects:

- With Italy, Spain and also France plus the UK having recorded high fatality numbers in 2020 – by mid-2020, the UK had the highest number of COVID-19 fatalities if any Western European country, namely around 40,000 while the highest fatality rates were in Belgium, Spain, UK, Italy, and France as the top five (weakest) countries with Sweden, Netherlands, Ireland, the US and Canada in rank 6–10 in the list of OECD countries – there are three weakened EU27 countries, plus Belgium as well as Sweden and the Netherlands where one may witness a lasting political trust shock so that political stability in the EU is weakened in a post-corona setting and this is bound to weaken the European Union as well. The US position in turn is also disappointingly weak as the Western world’s leading economy and this, along with the UK’s results, implies that the whole Western world is weakened from the corona shock in terms of political stability; moreover, western democracies seem to have weakened relative to countries with a more authoritarian regimes (China, possibly also Russia and Turkey).
- For western economies, the ability to cooperate could be weakened considerably as a consequence for the coronavirus epidemic shock. One can, however, not rule out that as a consequence of a weakening of certain Western European countries, EU integration might be reinforced provided that the EU’s reputation is less weakened than trust in national governments; as national governments were the key actors this is likely to be the case. However, it is not clear that a critical minimum of pro-EU integration countries will push for more EU integration or more political integration in the Eurozone. Since it is mainly younger voters who are losing trust in political institutions due to epidemic shocks, one should consider that ageing in Germany, Italy, Spain and Greece is progressing fast and in these countries the size of young voters’ cohorts is rather small so that the political impact might be modest in the end. The share of younger cohorts is larger in France (and in the UK) so that political instability there could become more of a problem in the medium term and populism – right-wing or left-wing – could play a larger role in the future which in turn would undermine Franco-German cooperation. In Germany, the health system and health policy as well as economic policy apparently have responded fairly well to the pandemic shock of 2020.
- With the US fairly weakened through the corona shocks, there is a Western leadership gap which in turn could bring more global political instability which would undermine global growth. The corona crisis was the first international economic crisis without US leadership; the EU is certainly incapable of replacing the US as the EU is much too weak. However, it could be a common interest of the EU, ASEAN and Mercosur to reinforce global stability by strengthening multilateralism and the role of international organizations.

## Policy conclusions

The analysis has shown that the Western world is facing a serious crisis as the populist policies of US President Donald Trump and the populist BREXIT project in the UK stand for an internationally destabilizing overlap – less free trade in the world economy; particularly as the Trump Administration has seriously undermined the WTO and other international organizations (e.g. WHO, World Postal Organization) and follows a protectionist trade policy, directed mainly against China, and a broad anti-multilateral policy agenda. The import tariffs envisaged in the UK for a No Deal case – i.e., no agreement between the EU27 and the UK about future trade relations – as well as the US import tariffs vis-à-vis China apparently are not in line with a modern optimum tariff literature, namely an approach which takes into account the role of cumulated outward foreign direct investment. As both the US and the UK are big source countries of FDI, the tariff rates envisaged for British imports from the EU27 and tariff rates imposed by the US on China, respectively, are too high and thus self-damaging. Moreover, such protectionism has negative welfare effects for other countries and the world economy, respectively. After the UK has left the EU, there will be a new EU-UK rivalry in third countries, including ASEAN which in turn might face a less stable EU; not least because of the pandemic’s political scar effects which could result in lasting political and economic destabilization of the EU27. The European Commission’s policy approach to fight the recession effects of the corona shock is not convincing and the combination with the second EU loan package from April 2020 – adopted by the European Council of Finance Ministers – is not even an effective hedge against a new Euro Crisis 2 which would have Italy as the epicenter. The unpragmatic crisis approach of Germany is a major problem as is a lack of willingness on the part of Italy to adopt a pro-growth program and other necessary systemic reforms which would stimulate innovation and growth. A “Joint Eurobonds” approach which would represent about €600 billion of a loan package of Eurozone countries – with 55% collateral from gold and reserves – would be needed to avoid a costly Euro crisis. The €750 billion package of the European Commission is not necessary, a much smaller transfer package of about €100 billion would be adequate where an emphasis on the efficient use of EU funds/transfers is required (see on efficiency aspects of EU structural funds: Becker et al. 2010). Major EU reforms as well as Eurozone reforms are necessary; the Eurozone should have a Eurozone Parliament since otherwise the adoption of an effective and efficient fiscal union complementing ECB monetary policy is not possible.

The corona shock undermines economic stability in the US, Europe and worldwide. The US suffers from a structural populism problem which in turn destabilizes international economic relations and which has also revealed a new US problem: An inability of the current US Administration – under President Trump – to exercise leadership in a major international economic crisis. This is a serious challenge for both the EU and ASEAN. Both groups, which could become victims of a new grand power regime dominated by the US, Russia and China, should try to combine their political and economic resources for a much broader cooperation; networked leadership via EU-ASEAN-Mercosur could become a new concept for maintaining multilateralism. New EU-ASEAN cooperation schemes for fighting epidemics and the Corona World Recession could be useful for both sides. One cannot rule out that US-Sino economic and political relations will be rather unstable for many years to come. Neither the EU nor ASEAN are interested in a Sino-US Cold War. The reform of the WTO is a major challenge for all countries. The argument of the Trump Administration that previous US negotiations were all concluded to the disadvantage of the US is not convincing at all – but Trump has ended NAFTA and now a new agreement under the name US-Mexico-Canada Agreement (USMCA) has been implemented. As regards the WTO, one should consider broader membership rights for regional integration clubs while unanimous voting should no longer be the rule for decision-making.

As regards financial stability in the world economy, there is a serious risk that the Corona World Recession will stimulate new financial market deregulation in the US and the UK which in turn would put pressure on EU countries to adopt new deregulation measures as well. Against such ill-advised changes, the G20 should reach an agreement on the long-term minimum principles of financial market regulation. Moreover, the IMF should, in the future, introduce an interdependency report both on stabilization policy and financial market regulations of major member countries and regional integration groups (e.g., EU, ASEAN, Mercosur, ECOWAS). As regards the US, it would be appropriate for policymakers to adopt major systemic reforms where health care systems in EU countries or Singapore, for example, could be inspiring for efficiency gains; universal health insurance in the US remains a political challenge and a rather different tax and social security system than currently may be required. States could implement their own social security system reforms, but a federal minimum requirement in certain fields could be useful.

### Two-stage international cooperation and IMF consistency issues in the Corona crisis

While the Corona World Recession calls for the global cooperation of countries facing a recession, one may argue that a joint IMF-World Bank initiative would be too broad and too complex as too many countries would be involved. While such an approach could ultimately be useful, the focus on the relative cost of cooperation and the benefits of joint stabilization policy suggest a different approach:

- An effective anti-corona pandemic policy could mean to first organize a consistent EU-ASEAN cooperation or a G20 cooperation and only later to establish a very broad joint stabilization initiative led by the IMF and the World Bank plus the WHO. A smaller group of countries will go along with more homogeneous political preferences and smaller transaction costs in the context of a rather limited number of countries. Hence a two-stage international coordination approach could be useful. Moreover, one may argue that isolated Article IV reports of the IMF are a doubtful exercise; at the very least, the the “big interdependency” between the US, the Eurozone, Japan and China should be analyzed in a consistent way. Here, both the real economy and financial market interdependencies could play a major role. It is unclear why the IMF’s Global Stability Report 2020 (IMF 2020c) does not mention the potential problem that a very high US deficit ratio in the corona crisis – as mentioned in the IMF’s Fiscal Monitor in mid-2020 – could bring about a strong downward rating of US government bonds which in turn could trigger parallel downgrades in dozens of other countries which both together could destabilize world financial markets and the world economy, respectively, strongly.
- Given the fact that the Corona World Recession has brought about a strong rise of protectionism in many forms in many countries, a special monitoring group of the WTO and the World Bank could be useful to bring down protectionism in the world economy.

There is – under Trump’s non-leadership in the international economic recession - the question as to the extent to which new leadership approaches could work in the Corona World Recession and in future international economic crisis situations.

### Networked leadership in the twenty-first century

Regional integration schemes could consider to create networked leadership inside and outside of international organizations. A cooperation between the EU and ASEAN could be of key importance in this respect. The ASEAN group has, however, a weak point, namely that it lacks adequate institutions for such political networked leadership; there is no ASEAN Commission and no ASEAN Parliament. To the extent that the US becomes increasingly unreliable as a political supporter and military ally for EU NATO countries, and for ASEAN countries as well, there could be the option for ASEAN countries to largely give in to the rising political and economic pressure of China and follow increasingly follow China’s political agenda. On the other hand, ASEAN countries could seek a stronger cooperation ASEAN-EU which indeed could indirectly be an impulse in stabilizing the US so that it regains its traditional leadership role. Shared leadership could be an important new concept in the twenty-first century and even cooperation among EU-ASEAN-Mercosur could be possible once Brazil no longer has a populist president. It is rather unclear to what extent and how fast ASEAN countries will upgrade its institutions; without a supranational institution – which could be similar to the European Commission – and that means without some transfer of competences to ASEAN as a political integration club, there is no opportunity for strong EU-ASEAN political cooperation. EU-ASEAN-Mercosur-ECOWAS cooperation would be a useful long-term platform for maintaining global stability and multilateralism, respectively. It seems natural that the EU would have to envisage not just more economic and political cooperation with ASEAN, but also enhanced medical cooperation in fighting future pandemics. Long before the coronavirus pandemic, many EU countries could have learned that Singapore’s health system is worthwhile to study as a benchmark.

As regards the transatlantic trade policy conflict, it could be useful to establish a trilateral expert group on global and regional international imbalances; emphasis on experts means that the high-level political conflicts that so far have played a considerable role should become weaker. It is unclear whether or not the US wants to continue its traditional role as a hegemon with willingness to redistribute benefits from trade liberalization in a global and multilateral context. Since China’s export-GDP ratio has started to decline considerably after 2010 the willingness of China to assume such a role at a global level could be rather modest which could be a serious problem for maintaining a global free trading system. The EU is hardly able to assume such a role – not only because its economy is smaller than that of the US and of China; also the willingness of other countries to follow the political strategy of the EU27 is quite limited (with the UK being an example of massive obstruction). One cannot exclude that an internally divided US will become a more inward-looking country for many years to come. It remains to be seen whether or not serious EU reforms are undertaken which could be a basis for defending multilateralism in a pragmatic networked approach; such an approach needs rules and active management plus a pool of resources for international compensation payments within a plausible framework. As for policymakers the final conclusion is: Much to do for everybody in the early twenty-first century, but at least it is the first century in which digital networking and algorithms create new options and opportunities for political actors and also for multinational companies. Those companies might become smaller in the future through more outsourcing and international offshoring so that foreign direct investment could gain importance – here one has to correct FDI-openness data for country size effects (smaller countries automatically are more open than big economies – see for some results on true FDI openness the appendix).

A special problem has occurred in the field of joint international infrastructure investments in the case of the Nord Stream II gas pipeline; for such ventures there should be a political clearing process in NATO before any such investment starts – rules similar to the WTO procedures should guide the decision-making. As regards potential “green import tariffs” of the EU designed to impair e.g. the import of steel produced with CO2-intensive technologies the economic logic suggests that such tariffs would be illegal if the exporting country (where production takes place) has a CO2-emission certificate trading system that is equivalent to that of the EU; China e.g. has introduced a CO2 certificate trade system in 2020 but the system is not equivalent to that of the EU since China will cover about 25% of CO2 emissions while the EU covers 45% of CO2 emissions; and manufacturing industry is not covered in China in the first stage of the new CO2 allowance trading system (and the share of CO2 emissions covered in California is around 80% which is higher than in the EU, but the rest of the US has only weak CO2 emission trading).. Clearly, this type of potential climate policy-related conflicts in trade should be considered in the WTO reform agenda. As regards EU-ASEAN there is clearly both a lack of joint research projects and a certain lack of political cooperation among key regions in Europe and Asia. China’s special links to the EU will be reinforced through a rapid rise of daily block trains – a unique link not existing for the USA.

The weakening of the US and the economic rise of China could make China’s government more assertive in ASEAN and Asia, respectively. Australia and Japan thus could become more interested in creating stronger military links to NATO and ASEAN countries might consider options in this direction as well – despite considerable sympathy amongst part of the populations of certain ASEAN countries in favor of China. The political conflicts over Hong Kong – with a strong intervention on the part of China’s government in the corona year 2020 and a visibly engaged UK government announcing its offer of simplified and expedited visa procedures and a path to citizenship for up to three million people in Hong Kong – in turn could reinforce political resistance in some Asian countries vis-à-vis China. If the US military presence in Europe should decline, EU countries could reinforce new steps to consider stronger cooperation in favor of joint defense in Europe/EU (picking up failed historical initiatives in the 1950s). The political economy of international economic relations will face new challenges in the early twenty-first century. Will the EU clearly signal to China not only which rules and restrictions, respectively, are relevant for inward foreign direct investment (April 2019) but also indicate how the EU-US cooperation could be reinforced in the medium term? Meanwhile, the US, having adopted a less friendly view on China, could support India in a broader approach much more than previously which in turn could reinforce China-India conflict areas. As regards the coronavirus vaccination challenge, both India and China could play a critical role for the world as India is one of the leading global producers of vaccines – at least until 2020.

## Figures and Tables

**Fig. 5:**
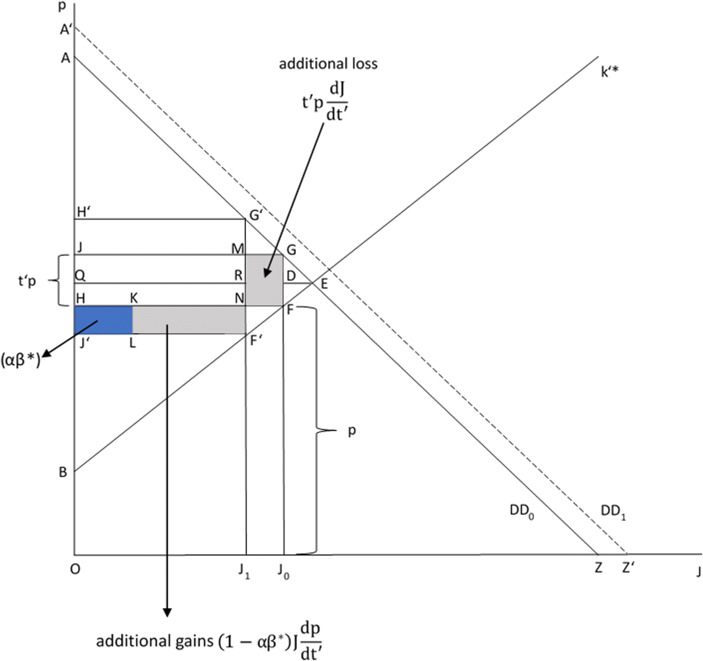
Optimum Import Tariff in Setting with Outward Foreign Direct Investment. Source: Own representation.

**Fig. 6:**
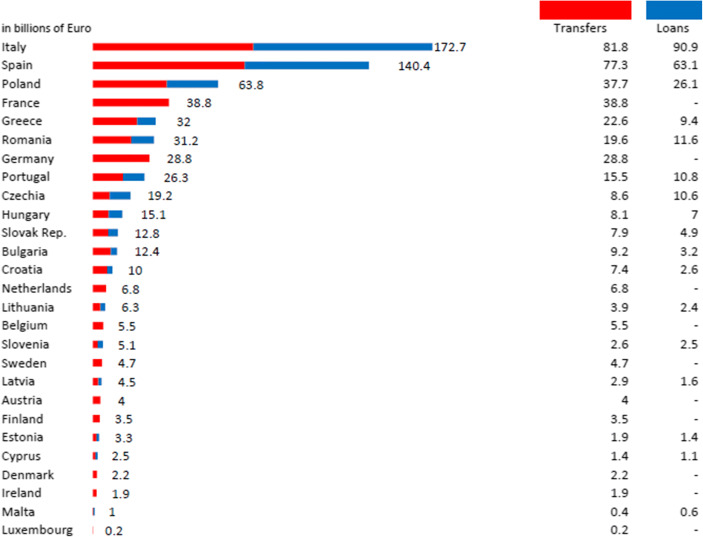
European Commission Proposal for a Post-Corona Package for EU Member Countries (ordered by transfers)

**Fig. 7:**
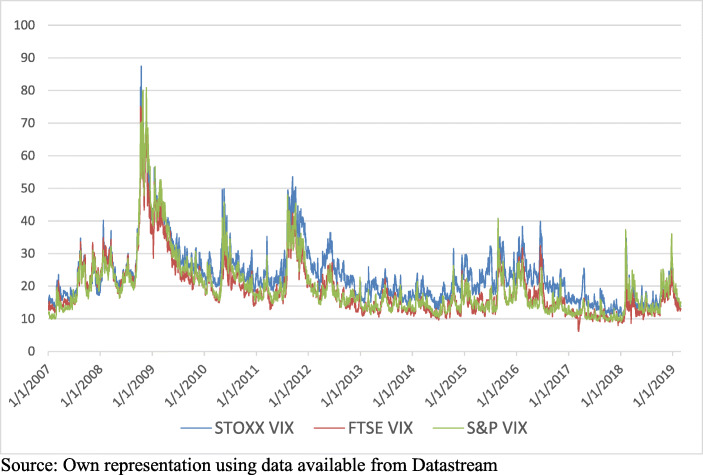
Implied Volatility Index for EA, UK and US (Daily, Jan. 2007 – Feb. 2019)

**Fig. 8:**
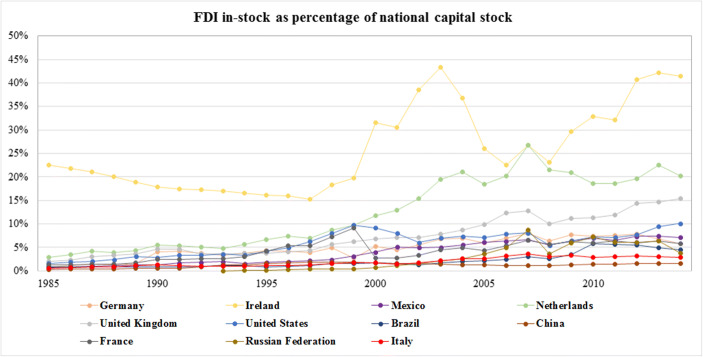
Foreign Direct Investment Ownership in Selected Countries. Source: EIIW calculations using data from UNCTAD

**Fig. 9:**
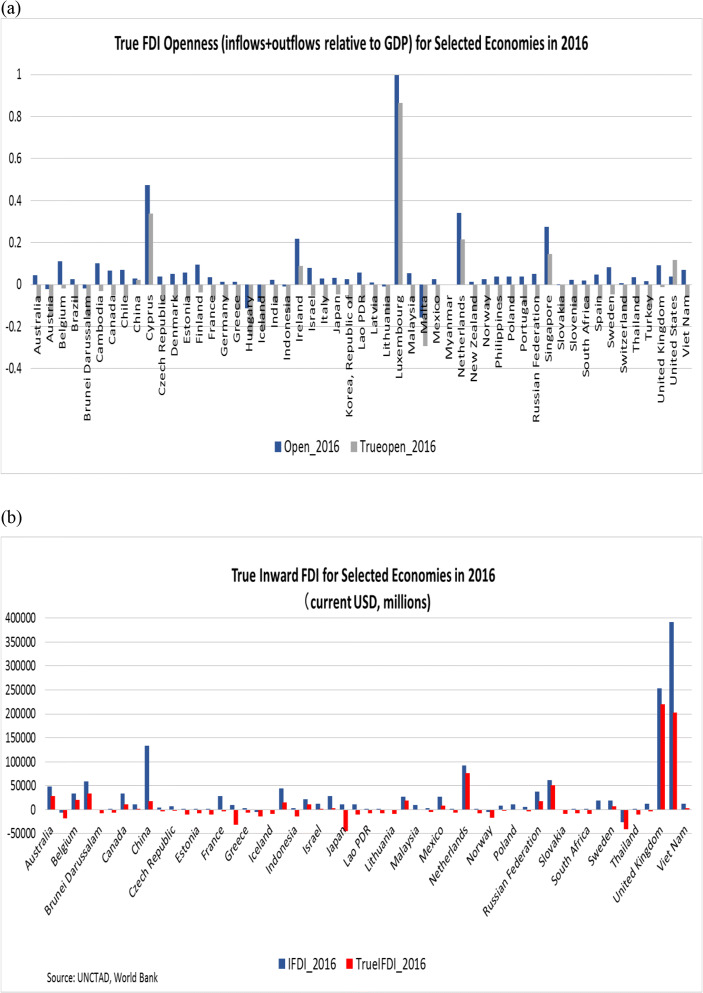

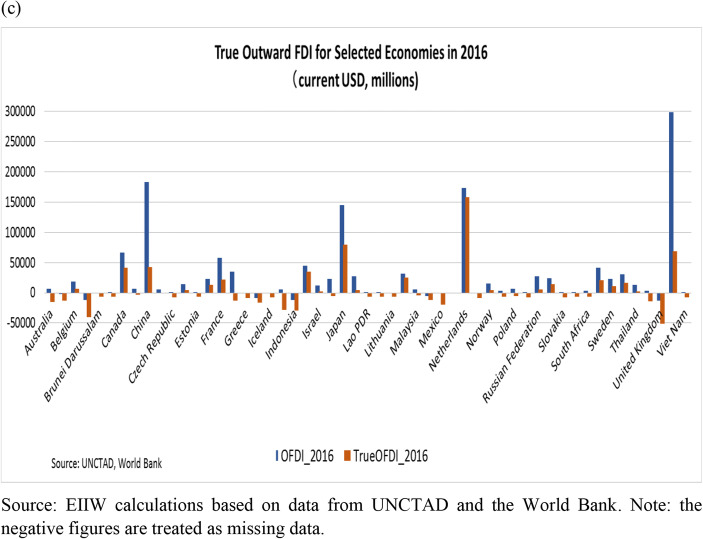
(a-c): Country-Size Corrected FDI Openness, FDI Inward Intensity and FDI Outward Intensity*. Source: EIIW calculations based on data from UNCTAD and the World Bank. Note: the negative figures are treated as missing data*

**Fig. 10:**
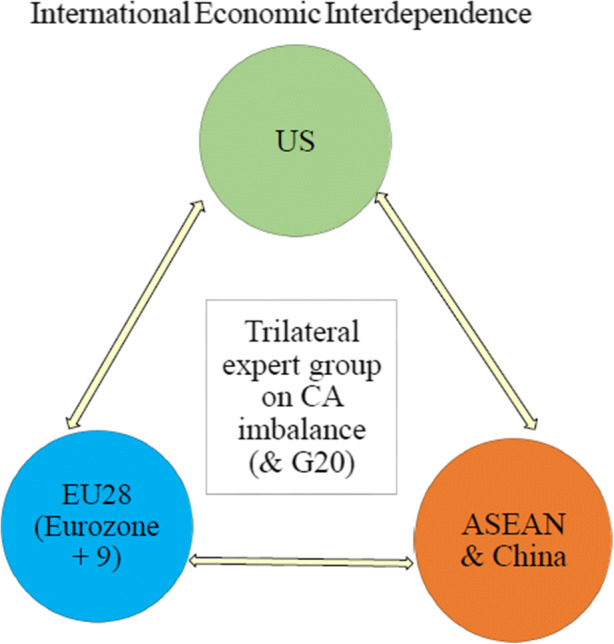
International Economic Interdependence. Source: Own representation.

**Fig. 11:**
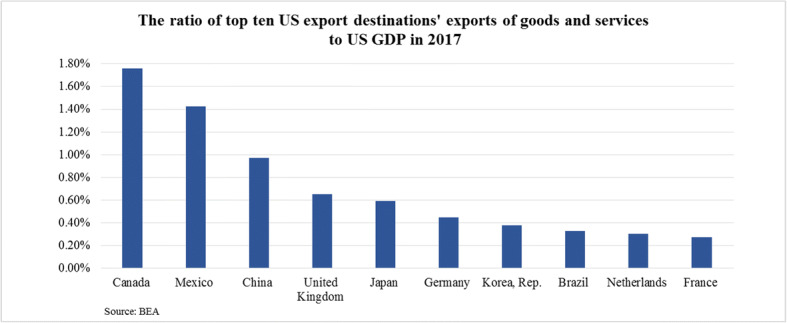
The Ratio of the Top Ten US Export Destinations’ Exports to US GDP (2017). Source: BEA

**Fig. 12:**
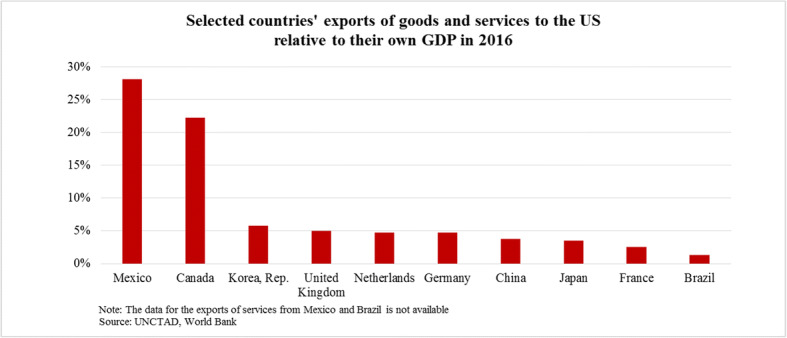
Selected Countries’ Exports of Goods and Services to the US Relative to Their GDP (2016). Source: UNCTAD/World Bank

**Fig. 13:**
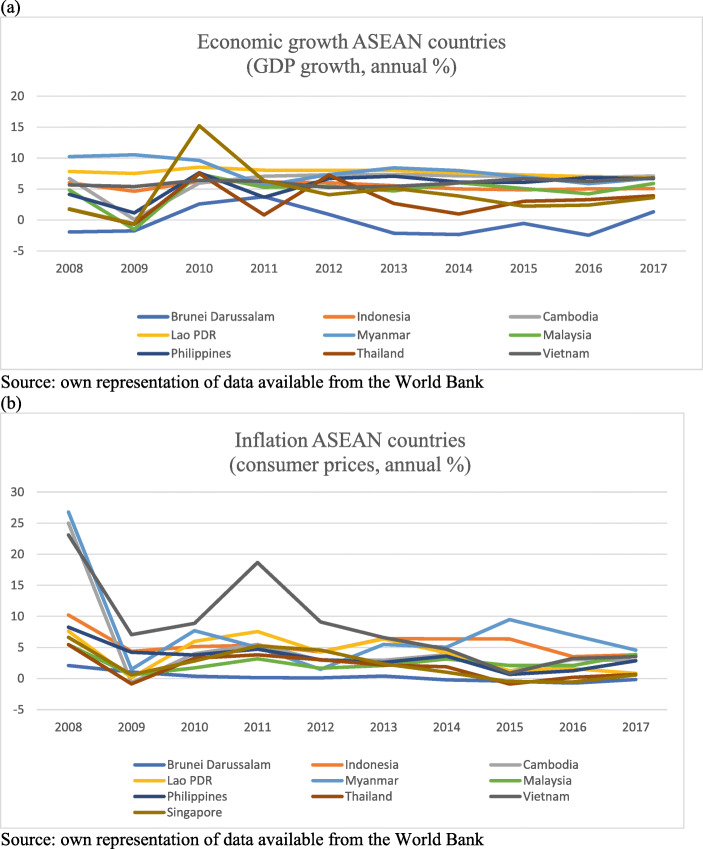

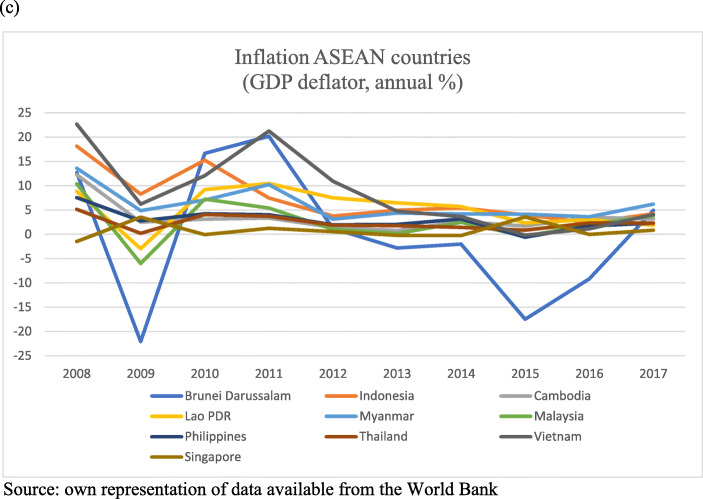
(a)-(c): Economic Growth and Inflation in ASEAN Countries, 2008–2017

**Table 12:**
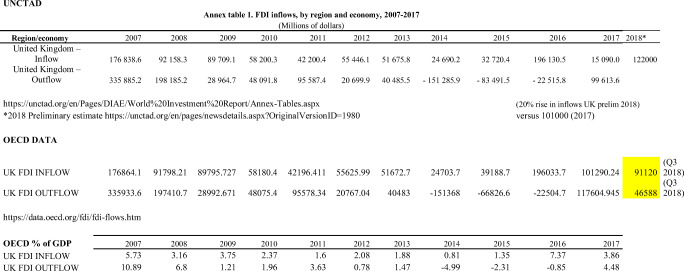
UK FDI Inflows and Outflows, 2007–2017

*Note: Figures in yellow relate to figures up to Q3 2018

**Fig. 14:**
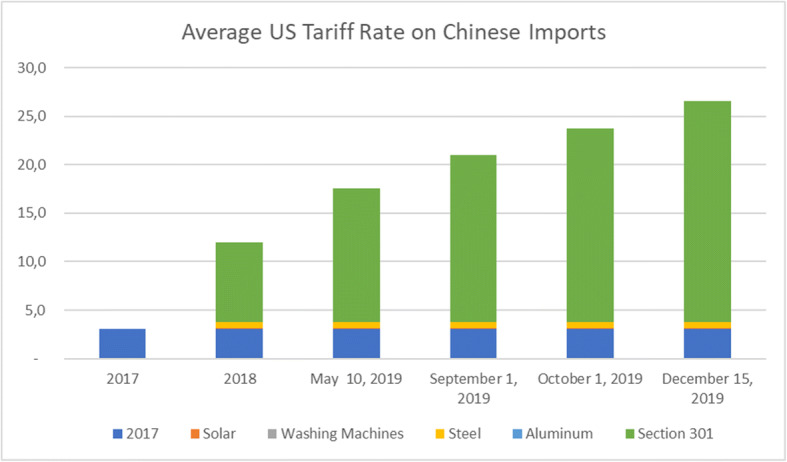
Average US Tariff Rate on Chinese Imports

## Appendix 1: The Optimum Tariff in the Presence of Outward Foreign Direct Investment

In Economics, the optimum import tariff of country 1 (home country) is shown to be 1/E’ where E’ is the foreign export elasticity (supply elasticity). If, however, there is outward foreign direct investment (FDI) in country 2, the situation is different as will be shown subsequently. Since cumulated outward foreign direct investment has increased considerably in OECD countries and Newly Industrialized Countries since the mid-1980s, it is important to consider the effects of outward FDI in the optimum tariff literature. The share of foreign investment also varies considerably by sector; as regards the necessary modification of the optimum tariff literature, one might argue that only outward FDI in the tradable sector is important, but additional reflections will show that outward FDI in the non-tradable sector could also be relevant. It is remarkable that the Trump Administration, in its trade conflicts with China, has emphasized that US import tariffs would be quite favorable for the United States and that high import tariff revenues would accrue to the US. However, the Trump Administration’s list of import tariffs vis-à-vis China has ignored any foreign direct investment effects although US multinational companies have relatively high investments in many sectors in China. Welfens (2019a, pp. 325–327) has pointed out this US policy inconsistency – with key aspects explained in a graphical approach. Subsequently, the simple mathematics of the modified optimum tariff solution, namely for the case of outward cumulated FDI, are presented.

The debate about import tariff and inward FDI has played some role in the literature, particularly as Bhagwati (2004) has argued that the presence of foreign investors and multinational companies, respectively, will create strong lobbying pressure in host countries to have low import tariffs – and this pressure should be the higher, the higher the share of intermediate imports relative to gross domestic products is. As regards the openness of countries with respect to trade and foreign direct investment, one may point out that for many economic aspects effective trade openness (Bretschger and Hettich 2002) is crucial where one has corrected the trade-GDP ratio for the size effect of countries. In a similar way, the following graph shows the true FDI openness of selected countries.

As regards FDI inflows in OECD countries, many crucial factors can be shown within a gravity modelling approach to influence FDI dynamics (Welfens and Baier 2018) and in a similar way outward FDI dynamics (Baier and Welfens 2019) show clearly the variables that are influencing FDI flows. While FDI flows have become increasingly important in the world economy since the 1980s, the impact of FDI on key economic variables has not been studied broadly, despite the fact that the role of multinational companies has been growing over decades in many countries.

In the subsequent analysis, the role of FDI for trade in intermediate products will not be analyzed. Section 2 presents the theoretical derivation of a new optimum tariff formula. Section 3 shows the crucial policy relevance of this new formula for both the United States, for example in the context of the US-Sino trade conflicts under the Trump Administration, and it is argued that the UK import tariff list for a No-Deal case with the EU – it might become relevant in 2021 – also contains import tariffs which are too high for an optimum tariff rate.

### Optimum Tariff in the Presence of Outward FDI: Theoretical Aspects

In the traditional optimum tariff literature, the case of a large economy typically is considered. The foreign exports are shown as the supply curve k’* (* for foreign variable, k’ is marginal costs). Import demand is given by the schedule DD0 in the sector considered. Import quantity is denoted by J, the foreign offer price net of the import tariff t’ is denoted as p. The optimum tariff will balance the additional gains which emerge from a reduced world market price (Jdp/dt’) – after the imposition of the tariff in country 1 – and the welfare loss from reduced import volume; both areas are shown in the subsequent figure.

1 $$ \frac{Jdp}{dt^{\prime }}-{t}^{\prime opt}\left(\frac{pdJ}{d{t}^{\prime }}\right)=0 $$

2 $$ {t}^{\prime opt}=\frac{\left(\frac{dp}{dt^{\prime }}\right)J}{\left(\frac{dJ}{dt^{\prime }}\right)p} $$

The reduction of the world market price through import tariffs in country 1 amounts to lower profits of the exporting country (country 2). As the supply elasticity of exports E’ is (dJ/dt’)/(dp/dt’)(P/M), the traditional result is given by:

3 $$ {t}^{\prime opt}=\frac{1}{E^{\prime }} $$

If, however, foreign investors own a share α in the capital stock abroad and all goods are tradables, the marginal gain from reduced profits abroad is – with ß* denoting the share of profits in gross domestic product abroad - given by the term (1- αß*) J(dp/dt’), so that welfare maximization is now given by the condition:

4 $$ \left(1-\alpha \beta \ast \right)\frac{Jdp}{dt^{\prime }}-{t}^{\prime opt}\frac{pdJ}{dt^{\prime }}=0 $$

Here it has to be considered that the import tariff reduces the exports of country 1 firms’ subsidiaries in country 2, and lower profits in subsidiaries abroad reduce overall profits of country 1 companies – this should dampen the stock market price index in country 1; lower profits in MNC subsidiaries also imply a lower real national income (Z) which is the sum of real GDP (Y) plus net income from abroad.

In the presence of outward FDI, the optimum import tariff for any sector (i; subscripts are dropped here) is now given

5 $$ t{'}^{opt}=\frac{1-\alpha \mathrm{\ss}\ast }{E'} $$

Accordingly, the presence of outward foreign direct investment reduces the optimum import tariff. If foreigners would own the whole capital stock so that α would be unity and if one assumes that ß* is 40% - as in many developing countries (e.g., in China) – or the familiar 1/3 in many OECD countries, then the optimum import tariff of a big OECD economy would be only 60% of the traditional optimal import tariff.

The Bhagwati conjecture that foreign direct investment should bring about less protectionism is reinforced by the analysis presented here – even if there is no trade in intermediate products. This in turn implies that regional integration schemes should be viewed with modest reservations provided that the (small) countries that create a regional integration club can be considered to be important source countries of outward FDI. As most Western EU countries were already major FDI source countries in the 1980s, the EU southern enlargement, the EFTA enlargement round in the 1990s and the eastern EU enlargement rounds after 2004 should be rather uncritical in terms of import tariff pressure. In this perspective, the UK – BREXIT implementation has started on January 31, 2020 – has been an important country for a rather pro-free trade policy stance in the EU (not just because of the long-standing UK tradition of free trade, but also because the UK has been a major source country of outward FDI in the EU).

In the reality of import tariff policy, one will have to consider various sectors (i = 1, 2…N) which will all have different supply elasticities. Typically, supply elasticities will be rather high in sectors which can be characterized by low-technology products. In contrast, the export elasticity of high technology products should have a rather low price elasticity unless the respective firms are obtaining a patent-based monopoly position which is a special aspect not considered further here – clearly, the monopoly firms (after having obtained a patent) will offer in the price-elastic part of the demand curve.

An analysis with a focus on certain individual sectors is not a macroeconomic approach, but one could easily integrate macro aspects in the analytical framework presented here if a broad trade policy with generally rising import tariff is considered:

- If country 1’s government imposes import tariffs on all or many sectors of a big trading partner, the macroeconomic effect in country 2 will be a decline of real gross domestic product. Thus, real imports from country 2 will reduce which, in turn, will dampen the real GDP of country 1 whose net exports of goods and services will decline.
- It is noteworthy that aggregate consumption C is a positive function of real national income Z = Y + αß*Y*q* where q* is the real exchange rate; here asymmetric FDI is considered, so that only MNCs from country 1 are investors abroad (in addition, two-way FDI could also be considered: Welfens 2011). Not only is C proportionate to real disposable national income ((1-t”)Z) – with t” denoting the income tax rate - but also the demand for goods imported in general and for imported goods in individual sectors. If import tariffs are imposed by country 1 on many export sectors of country 2, then Y* will decline and therefore (at a given real exchange rate) Z will decline and this in turn implies a downward shift in all import markets and actually also in all consumption markets. This further adds a negative welfare effect on country 1 so that the optimum import tariff rate will be smaller than in the modified new formula presented above.
- It should be noted that the negative welfare effect of outward FDI for country 1 would be smaller than shown in the above new equation for the modified optimum import tariff, if part of the economy abroad consists of a non-tradables sector and if indeed part of outward FDI of country 1 firms went to the non-tradables sector in country 2. However, the negative macroeconomic effect discussed would still be relevant for an enhanced welfare analysis.

It is clear that an approach with inward and outward FDI would require to change the definition of Z to become Z = Y(1-α*ß) + αß*Y*q*. This in turn would be an adequate framework to study the problems of an international tariff war, but clearly that scenario would be more complicated than the rather simple setup presented here. However, this rather compact setting has already shown clear implications for policymakers, including for the Trump Administration in the US and the Johnson government in the United Kingdom post-BREXIT – with both governments so far lacking an adequate optimal import tariff policy.

The optimum tariff analysis is related to the Marshall Lerner condition and the enhanced (sharper) Marshall Lerner condition (Welfens 2019b) which takes into account the role of foreign direct investment. Clearly, an optimum sectoral import tariff will reduce the expenditures for imports in a given sector and broad application of optimal import tariffs across sectors will in turn affect both imports as well as real national income. If the application of the optimum import tariffs brings about a real appreciation of the currency of country 1 – hence a depreciation of the currency of country 2 –, there is a further macroeconomic effect: As the Froot and Stein (1991) arguments suggest that a real depreciation in a world of imperfect capital markets will go along with higher FDI inflows, multinationals from country 1 will invest more in country 2 in the setting with asymmetric FDI considered above at first. In a two-way FDI setting, both higher outward FDI from country 1 and lower inward FDI from country 2 have to be considered – and the associated shifts in market demand curves in the tradables sector.

The Trump Administration has adopted a rather aggressive tariff policy vis-à-vis China in 2018/19; only in January 2020 has a first US-China agreement in trade policy been adopted. As regards the US-Sino trade conflict under President Trump, one may assume that the Trump Administration has chosen import tariffs for Chinese exporters in line with the traditional optimum tariff literature. Since the US and US firms are important FDI sources for most export sectors in China it is clear that the Trump Administration has adopted excessive import tariffs where it has not been considered that profits of US subsidiaries in China will be reduced by US import tariffs. This in turn would partly explain why the US stock market indices have reacted negatively to US import tariffs.

It is also noteworthy that the UK under the May government has already released a list of intended import tariffs for the potential case of a No-Deal BREXIT (see appendix). To the extent that the UK government has chosen import tariffs for the No-Deal scenario on the basis of the traditional import tariff literature, the proposed import tariffs are too high. This implies, of course, that the tariff revenues expected for the UK could be somewhat lower than considered so far in the literature; for example, the Institute for World Economics (IfW, Kiel) has calculated that the UK could, under a No-Deal scenario, expect import tariff revenues that would equal about €6 billion (Felbermayr et al. 2020).

In the US-China tariff conflict under President Trump, the FDI-related aspects have been ignored – and similarly in the UK with respect to No-Deal tariffs vis-à-vis the EU27 - so that import tariffs imposed/announced in several sectors are too high. While it should not be difficult to calculate optimum import tariffs in an enhanced approach with outward foreign direct investment, one should also be aware that import tariffs are not likely to generate considerable medium-term benefits for a big economy in a system of flexible exchange rates. To the extent that higher import tariffs transitorily improve the current account balance in the medium term, one should not overlook that an appreciation of the currency will make import goods relatively cheaper while exports become less profitable – with profits expressed in domestic currency units. This well-known foreign exchange rate argument from Corden (1987) also suggests that aggressive US import tariffs will not generate much benefit for the United States.

Moreover, to the extent that a rather ad hoc trade policy of the Trump Administration undermines confidence in US economic policy, the tariff policy of that administration could indeed indirectly dampen global output growth and thereby the prospects for higher US net exports of goods and services. One should not overlook that part of the US economic expansion in the years 2018–2019 was not due so much to a successful US trade policy – as emphasized by President Trump – but rather to a strong fiscal stimulus in the form of a strange expansionary fiscal policy in an economic upswing: with deficit-GDP ratios of 4% to 5% in 2018/2019; if such policies would be continued, the Congressional Budget Office (CBO 2020) expects a US debt-GDP ratio of 180% by 2050 which is such a negative US outlook that one may think that voters will stop a continued strange fiscal experiment in the US.

## Appendix 2: EU Budget and the Corona Challenge

Time for reforms in the EU is scarce. The rise of China and the challenge of US populism give the EU a crucial strategic role in the future for Europe and the world economy. The fact that the EU countries could not come up with quick consensus on the new seven-year EU budget in early 2020 indicates that, despite the importance of a clear signal to the Community and the rest of the world, that post-BREXIT the EU27 is still not able to achieve quick compromise. Beyond the new field of climate neutrality policy – the European Green Deal – the new European Commission of President von der Leyen seems to need more time to adopt a new convincing policy course. The new European Commission picked up the Franco-German EU loan initiative suggesting €500 billion as a multi-year extra funding package, possibly with large transfers to EU countries hit heavily by the coronavirus pandemic. As the Commission unveiled its proposal it turned out that the envisaged recovery budget was even bigger, namely €750 billion where all EU countries should get certain transfers. The EU was expected to take loans in international capital markets, but not in the form of Eurobonds, instead each EU member country would face a liability in line with its share of the EU’s gross domestic product. The idea of large transfers in favor of Italy, Spain, Poland and France seems to stand for historical political bargaining: Rather weak governments in Italy and Spain would obtain large transfers in the corona shock period so that populist forces in these countries (and in France) would be weakened.

Source: Own representation.

## Appendix 3: UK Imports and Exports of Goods and Services, 2016

**Table 6:** UK Imports of Goods and Services in 2016

	in Thousands GBP	in % of UK GDP
Germany	74,255,231.52	3.78%
United States of America	71,685,352.62	3.65%
China	45,642,855.75	2.32%
Netherlands	41,087,528.8	2.09%
France	38,578,733.98	1.96%
Spain	28,840,545.61	1.47%
Belgium	25,970,640.23	1.32%
Switzerland	23,206,506.57	1.18%
Italy	22,949,738.98	1.17%
Ireland	20,566,268.58	1.05%
EU 27	319,549,668.4	16.28%

Source: EIIW representation of data available from the International Trade Center Trade Statistics

**Table 7:** UK Exports of Goods and Services in 2016

	in Thousands GBP	in % of UK GDP
United States of America	97,347,748.89	4.958%
Germany	48,293,861.84	2.460%
France	33,306,334.76	1.696%
Netherlands	31,117,694.63	1.585%
Switzerland	26,920,656.2	1.371%
Ireland	26,293,230.01	1.339%
Italy	16,888,888.95	0.860%
China	16,696,970.56	0.850%
Belgium	15,598,175.03	0.794%
Spain	14,520,394	0.740%
EU 27	232,602,612.6	11.847%

Source: EIIW representation of data available from the International Trade Center Trade Statistics

## Appendix 4: Eurobarometer Polls, Image of the European Union, 2016 and 2018

Political support for EU integration has improved in 2018 compared to 2016 as the EU survey results show.

**Table 8:** Eurobarometer Polls; Spring 2016 and Autumn 2018, Image of the European Union

		**Total Positive**		**Change**		**Neutral**		**Total Negative**		**Change**		**Don’t Know**	
		**2016**	**2018**	**16/18**		**2016**	**2018**	**2016**	**2018**	**16/18**		**2016**	**2018**
**EU28**		**34**	**43**	**+**	**9**	**38**	**36**	**27**	**20**	**–**	**7**	**1**	**1**
Belgium	BE	35	41	**+**	6	33	41	31	18	**–**	13	1	0
Bulgaria	BG	51	56	**+**	5	30	23	17	17		0	2	4
Czechia	CZ	26	28	**+**	2	40	40	34	32	**–**	2	0	0
Denmark	DK	34	48	**+**	14	42	36	23	15	**–**	8	1	1
Germany	DE	29	47	**+**	18	41	37	29	15	**–**	14	1	1
Estonia	EE	33	45	**+**	12	47	45	17	9	**–**	8	3	1
Ireland	IE	58	64	**+**	6	27	28	14	8	**–**	6	1	0
Greece	EL	16	25	**+**	9	33	39	51	35	**–**	16	0	1
Spain	ES	30	43	**+**	13	44	43	23	13	**–**	10	3	1
France	FR	36	34	**–**	2	33	38	29	27	**–**	2	2	1
Croatia	HR	37	39	**+**	2	43	42	19	18	**–**	1	1	1
Italy	IT	32	35	**+**	3	38	36	27	27		0	3	2
Cyprus	CY	27	36	**+**	9	32	40	41	23	**–**	18	0	1
Latvia	LV	31	42	**+**	11	49	47	18	9	**–**	9	2	2
Lithuania	LT	43	48	**+**	5	47	45	9	6	**–**	3	1	1
Luxembourg	LU	45	56	**+**	11	32	26	22	18	**–**	4	1	0
Hungary	HU	33	43	**+**	10	41	38	25	19	**–**	6	1	0
Malta	MT	41	43	**+**	2	43	43	13	10	**–**	3	3	4
Netherlands	NL	33	46	**+**	13	38	37	29	16	**–**	13	0	1
Austria	AT	32	40	**+**	8	30	37	37	22	**–**	15	1	1
Poland	PL	47	54	**+**	7	37	36	15	10	**–**	5	1	0
Portugal	PT	41	53	**+**	12	39	34	18	12	**–**	6	2	1
Romania	RO	42	52	**+**	10	43	37	14	10	**–**	4	1	1
Slovenia	SI	32	38	**+**	6	46	43	20	18	**–**	2	2	1
Slovakia	SK	30	33	**+**	3	43	49	26	17	**–**	9	1	1
Finland	FI	33	40	**+**	7	44	44	22	15	**–**	7	1	1
Sweden	SE	36	53	**+**	17	38	33	26	14	**–**	12	0	0
**United Kingdom**	**UK***	**31**	**43**	**+**	**12**	**31**	**29**	**36**	**27**	**–**	**9**	**2**	**1**

Source: EIIW calculations based on European Commission’s Standard Eurobarometer 85, Spring 2016 and Standard Eurobarometer 90, Autumn 2018

*implied referendum result on the basis of Total Positive/(Total Positive+Total Negative)

2016: 46%: 54%; by comparison the EU Referendum on 23 June 2016: 48.1% pro-EU (Remain): 51.9% pro-Brexit (Leave); 2018: 61%:39%.

## Appendix 5: Implied Volatility Index for the Eurozone, UK and US, 2007–2019

Source: Own representation using data available from Datastream.

## Appendix 6: Foreign Ownership of the Domestic Capital Stock

The share of foreign ownership in the capital stock differs across countries as is obvious from the subsequent figure which portrays results for selected countries. It is rather surprising – a first sight – that Italy as one of the big EU countries has witnessed rather small cumulated FDI inflows relative to gross domestic product over many years. The UK, France and Germany are more internationalized in terms of FDI dynamics. China’s inward FDI stock as a share of the total capital stock in China has increased considerably over time. The equivalent share in Ireland – as a small open economy – is particularly high; however, there could also be a bias in the Irish and Dutch figures since Ireland and the Netherlands are considered to be low-tax havens for foreign investors; and part of inward FDI could be earmarked for outward FDI in other countries. Beyond these aspects, it could be useful to correct the data for a country-size effect and here one can indeed implement a regression for a sample of countries to be considered and the residuals then can be used to measure true FDI (or true trade openness). The measure of true openness naturally will depend on the sample of countries included; and in the case of FDI one will also have to consider problems with zero or negative entries in some countries – but a solution to deal with these challenges can be found.

The share of foreign ownership in the capital stock or of the inward FDI stock relative to GDP will depend on several influences; the size of the country could be one aspect: Smaller countries might have a share of foreign ownership which is always larger than in big economies so that it would be adequate to consider a size corrected measure of FDI multinationalization. In the subsequent figures, the results of the calculation of the size-corrected ownership of FDI globalization are indicated. It should be noted that Bretschger and Hettich (2002) came up with the idea of a size-corrected measurement of trade openness and a similar logic is applied here – for the first time – to foreign direct investment. Based on the concept of the calculations regarding true openness from Bretschger and Hettich (2002), the foreign direct investment true openness is the residuals from regression of size on normal openness, using the estimation procedure of panel-corrected standard errors:

$$ {Openness}_i={SIZE}_i+{\epsilon}_i $$

$$ {True\ openness}_i={Openness}_i-{\epsilon}_i $$

$$ \mathrm{Here},{Openness}_i={\left( FDI\  inflows+ outflows\right)}_i/{GDP}_I $$

$$ {SIZE}_i=\frac{GDP_i}{\overline{GDP}}\ast 100 $$

whereby, *i* refers to country (*i* = 1, … n).

A brief remark on risk is adequate since policy intervention in the US, Asia or Europe could raise risk in financial markets in various ways. The implication in the enhanced neoclassical growth model developed clearly is that long-run equilibrium output is negatively affected by risk. The steady state solution (#) for y’:= Y/(AL) can be written – with m’:= (M/P)/(AL) – in a rather compact way where a simple savings function will be used: S depends on disposable income and is also a positive function of the value-added tax rate τ’; τ is the income tax rate, τ’ the VAT rat; n is the exogenous growth rate of the population, a is the quasi-exogenous growth rate of knowledge, δ the rate of private capital depreciation, c and c’ are positive parameters). The enhanced neoclassical growth model leads (with A(t) = A_0_e’^at^ where e’ is the Euler number and t the time index, A is the initial level of knowledge) for an economy with a savings function S = s(1-τ)(1 + c’τ’)Y to a steady state capital intensity k’#:

$$ \left(\mathrm{VI}\right)\ \mathrm{k}'\#={\left\{\left(\mathrm{s}\left(1-\uptau \right)+\mathrm{c}'\uptau '\right)\left[\left(1+\upvarphi '\mathrm{x}\right)\left(1+\upvarphi "\mathrm{j}\right)\ \left(1-\upsigma '\upsigma \right)\mathrm{m}'{}^{\mathrm{\ss}'}\right]/\left(\mathrm{a}+\mathrm{n}+\updelta \right)\right\}}^{1/\left(1-\mathrm{\ss}\right)} $$

Taking into account the production function, we get for y’# (y’:=Y/(AL)):

$$ \left(\mathrm{VII}\right)\ \mathrm{y}'\#=\left[\left(1+\upvarphi '\mathrm{x}\right)\left(1+\upvarphi "\mathrm{j}\right)\ \left(1-\upsigma '\upsigma \right)\mathrm{m}'{}^{\mathrm{\ss}'}\right]. $$

$$ {\left\{\left(\mathrm{s}\left(1-\uptau \right)\left(1+\mathrm{c}'\uptau '\right)\right)\left[\left(1+\upvarphi '\mathrm{x}\right)\left(1+\upvarphi "\mathrm{j}\right)\ \left(1-\upsigma '\upsigma \right)\mathrm{m}'{}^{\mathrm{\ss}'}\right]/\left(\mathrm{a}+\mathrm{n}+\updelta \right)\right\}}^{\mathrm{\ss}/\left(1-\mathrm{\ss}\right)}. $$

$$ \left(\mathrm{VIII}\right)\ \mathrm{y}'\#=\left[{\left(1+\upvarphi '\mathrm{x}\right)}^{1/\left(1-\mathrm{\ss}\right)}\ {\left(1+\upvarphi "\mathrm{j}\right)}^{1/\left(1-\mathrm{\ss}\right)}\ {\left(1-\upsigma '\upsigma \right)}^{1/\left(1-\mathrm{\ss}\right)}\ \mathrm{m}'{}^{\mathrm{\ss}'+\mathrm{\ss}/\left(1-\mathrm{\ss}\right)}\right]\left\{\left(\mathrm{s}\left(1-\uptau \right)+.\mathrm{c}'\uptau '\right)\right]/\left(\mathrm{a}+\mathrm{n}+\updelta \right)\Big\}{}^{\mathrm{\ss}/\left(1-\mathrm{\ss}\right)}. $$

Taking logs gives – while assuming φ’x, φ”j and σ’σ to be close to zero so that the approximation ln(1 + x’) ≈ x’ can be used – then for lny (with ß”:=1/(1-ß) and defining the non-deprecation rate of capital δ” =1-δ and assuming that a + n-δ is close to zero; A_0_ is the initial level of knowledge and t the time index; y:=Y/L; m’:=(M/P)/(AL), m:=(M/P)/L):

(IX) lny ≈ ß”φ’x + ß”φ”j - ß”σ’σ + (ß’ + ß/(1-ß))lnm + (ß/(1-ß))lns – ß/(1-ß)τ.

+ ß/(1-ß)c’τ’ – (ß/(1-ß))(a + n - δ”) + (1+ ß’ + ß/(1-ß))lnA_0_ + (1 + ß’ + ß/(1-ß))at.

This formulation suggests that traditional empirical growth analysis which ignores m’ as a production factor overestimates the technological progress rate a since the term (1 + ß’ + ß/(1-ß)) should not be ignored. In the analysis derived here the level of the growth path of per capita income is:

1. a positive function of the export intensity x
2. a positive function of the import intensity j
3. a negative function of the financial market volatility σ
4. a positive function of per capita real money balances m
5. a positive function of the savings ratio s
6. a negative function of the income tax rate τ
7. a positive function of the VAT rate τ’
8. a positive function of the term (a + n - δ”)
9. a positive function of initial level of the growth path lnA_0_

It is also noteworthy with respect to the steady state growth rate of per capita income (see the term (1 + ß’ + ß/(1-ß))at) that:

- The progress rate a has an impact on the steady-state growth rate – as usual.
- In addition: The long run growth rate is not simply determined by a; but also positively influenced by the output elasticity (ß’) of real money balances per worker in efficiency units and by the output elasticity of capital.
- If we take into account the progress function/knowledge production function (IV) (which makes the progress rate a quasi-exogenous variable in the enhanced neo-classical production function), we can also see the influence of the foreign direct investment variable α* and of j (for empirical evidence for the EU countries see Jungmittag and Welfens 2016). Note, if the hypothesis were that it is not only α* which has an impact on the progress rate a but also α, one would have to also consider also the role of cumulated outward FDI for the growth rate of knowledge.

From this theoretical perspective it holds: Protectionism will reduce the level of the growth path through 1) and 2) and also through 3) if there is an increase of financial market volatility through international tariff warfare which undermines the stability of expectations of future profit rates and the stock market price index development, respectively. Protectionism could also undermine the growth rate of knowledge if there is a negative impact on (cumulated) foreign direct investment.

This compact analysis is largely in line with what Mr. Carney, Governor of the Bank of England, pointed out with respect to the Trump-initiated tariff war on July 5, 2018 (Financial Times 2018, p. 2: BoE’s Carney warns of hit to global growth from all-out trade war): A 10% increase by the United States and its trading partners could, under a worst case scenario, slow down the US real gross domestic product by about 5% over three years. The direct effects of the tariff increases already announced by the US and various other countries could be largely confined to these countries, however, the Bank of England’s model suggests a worldwide impact if the US and all its trading partners would increase import tariffs by 10 percentage points: The US real GDP would fall by 2.5% and global output would fall by 1% simply through trade channel effects. Carney expressed a warning about a global trade war - if global confidence fell, financial conditions tightened and the tariffs were viewed as permanent, this could plausibly double the real GDP losses; and in the longer run output losses would increase due to the fact that lower trade openness would dampen productivity growth. With respect to BREXIT and the UK government’s Global Britain approach, Carney remarked:

*“As we know, the intention of Brexit is not to turn inwards, but to broaden openness over time…But Brexit will, for a period, be an example of de-globalisation because any reduction in openness with the EU is unlikely to be immediately compensated by new ties of a similar magnitude with other trade partners.”*

In a theoretical context, the Trump complaints and the Trump-initiated global trade war as well as BREXIT raise interest regarding the impact of the real exchange rate – and hence of shifts in international relative prices – on the trade balance and the current account, respectively.

## Appendix 7: EU Reform and Overcoming Populism in the World Economy?

From a global perspective, there are two key strategic questions in the early 21st century. Will the EU27 – post-BREXIT – generate sufficient adequate reforms to stabilize the European Union and can Western populism be reversed? There is a strong need to reform the EU in the sense that without reforms the European Community is likely to disintegrate, but the Political Economy of EU reforms is complicated and can be summarized as follows:

With BREXIT the EU faces new degrees of freedom to consider more integration in the field of military cooperation – here the UK had blocked EU initiatives over many years as the UK did not want to have rival structures to NATO emerging (the relevance of NATO has also diminished due to the lack of clear orientation of the Trump Administration).

- There is rising populism in many EU countries which means that a transfer of policy fields or budget funds to the supranational policy layer would be a difficult issue with these countries.
- The Eurozone is not really stable since the problem of fiscal responsibility has not been really solved – and with Italy having a populist government, the ability of the Eurozone countries to achieve sensible rules for government debt limits are rather limited.
- It should not be overlooked that the EU (European semester) and the IMF (Article IV mission) have all kind of short-term monitoring of EU countries’ development, but the equally important monitoring of long run growth has been largely neglected and this is a serious shortcoming in policy monitoring: In Italy real disposable per capita income of 2006 was not higher than in 1995 and no systematic action was taken against this critical long run quasi-stagnation.
- As long as Germany and France, plus Spain and other Euro countries in central Europe, push for similar reforms in the Eurozone there could be a chance to make progress in institution reforms of the Eurozone.
- As regards the power of EU countries in the Council of Ministers – with weighted voting – BREXIT will raise the power of big countries; the relative power increase is largest for Spain and Poland if one considers the Banzhaf power index (Kirsch 2016).

As regards populism in the Western world, one has to consider the situation in the US as the leading country of the West, namely the US, and the dynamics in some other countries. The prospects for stopping US populism are not good in the medium term if one accepts the hypothesis that the constantly declining relative income position of the lower half of the income pyramid and the “political noise of wishful thinking” – often pushed by digital social networks - was behind the election of Donald Trump in 2016:

- The ongoing expansion of the digital economy in the US will reinforce the increase of the income share of the top 10% and reduce further the share of the lower half of the income pyramid. Students’ tuition fees in US universities have strongly increased after the US banking crisis and hence the opportunities for the young generation to get enhanced human capital formation are declining. This is critical in a period in which the share of unskilled workers’ jobs in US manufacturing industry is declining (Lawrence 2017).
- If a hard BREXIT is implemented the UK would become Western Europe’s largest populist economy where government promises benefits from a disintegration project that certainly will bring a major loss of income growth in the medium term in the UK. A populist UK government – which might witness a second Scottish Independence Referendum – would be the vassal of the US and both would stand for a populist duo that should be expected to push for nationalist populist political expansion in EU27 countries.
- The expansion of populist political parties in EU27 would contribute to further EU disintegration which in turn could set a disintegration model for other regional integration clubs in the world economy.
- If the US populism would continue the role of International Organizations would considerably reduce and a new global system of Great Powers – the US, China and Russia (plus possibly the EU) –would shape the world economy; higher military expenditures relative to GDP and more military conflicts worldwide could be the consequence, coupled with more international migration which in turn could reinforce political populism.

The main challenge of an amateurish US trade policy is that a protectionist Trump approach will weaken economic globalization and hence the catching-up process in the South that normally could be expected from ongoing globalization. This in turn would reinforce migration pressure to the North and hence would nurture further populism. One should also note that Africa’s general economic catching-up process is not likely to simply reduce the emigration pressure from Africa towards Europe and Asia. Research has shown (Clemens and Postel 2017) that a rising per capita income typically will enhance the ability to emigrate – only if a critical per capita income y## is reached will emigration pressure reduce.

If the EU and Asia do not want to become the victim of the inconsistent and growth-dampening Trumpian trade policy there is no other way than to organize an international containment policy against the Trump Administration. Since Trump is likely to use the whole economic arsenal to force other countries to follow the US lead it will be important to have the role of the Euro reinforced as an international currency or alternatively to seek a global political compromise with the US in the G20. Trump would find his position uncomfortable with respect to his political constituency if the US would be rather isolated in the G20 (and the G7). If the EU wants to be more independent on the US the EU will have to invest more in common defense projects.

As regards EU cooperation with ASEAN there are some opportunities. The EU is the No. 1 foreign investor in the ASEAN. If there would an EU-ASEAN free trade agreement, the prospects for growing EU-ASEAN trade would improve considerably. ASEAN still could increase intra-ASEAN-trade as the share of this trade is rather low. A careful system of FDI incentives could be useful for raising intra-ASEAN trade.

The EU and ASEAN should create an economic policy management framework that considers different US policy actions (or Chinese policy actions) and the appropriate policy responses to minimize risk for the EU economy and the ASEAN economy, respectively. It could be a useful political hedging strategy for the EU and ASEAN if there would be an EU-ASEAN free trade and investment treaty so that barriers to foreign direct investment could be reduced alongside embracing free trade. Bilateral free trade agreements between the EU and individual ASEAN countries can be a useful transitory policy element, but given the fact that ASEAN will have all 10 member countries in the ASEAN single market, it would make considerable sense to have an EU-ASEAN single market integration framework. EU-ASEAN foreign direct investment perspectives could be greatly broadened – the EU is No. 1 in ASEAN countries; many young multinational firms from ASEAN have not yet explored the rich investment opportunities in the European Union. Enhanced international cooperation between the EU and ASEAN in many fields of innovation and entrepreneurship could be created within modernized policy cooperation approaches between the EU and ASEAN.

From the perspective of future EU-ASEAN developments, further dialogue and cooperation in the field of economic policy and social policy, with a view to contributing to sustainable and inclusive growth, social cohesion, and labour market stability including decent work and core labour standards, should be encouraged. Furthermore, work towards the timely resumption of the ASEAN-EU Free Trade Agreement (FTA) negotiations should be intensified, taking into consideration the status of bilateral FTAs between several ASEAN Member States and the EU. Both the EU and ASEAN should jointly explore ways to develop transparent, coherent, ICT regulatory framework as well as other ICT priorities.

As regards future comparative research, one should look at both trade and FDI in the EU-ASEAN perspective; on top of this, one should take into account migration issues which have already been studied in a gravity model approach by Tuccio (2017). As regards the EU–ASEAN Action Plan 2018–2022 (ASEAN 2018), it seems that a narrower list of key points could be more useful than the current very long list of policy suggestions.

## Appendix 8: Weakening Multilateralism – Towards a Great Power Regime?

Multilateralism means a rule-based international system with a crucial role for International Organizations that help to implement international law. In the words of the Secretary General of the WTO – as expressed in a speech in 2017 – multilateralism means “to make the small big and the big civilized”. The idea is that big powers that have tied their hands to the rule of international law would be a fairer partner for the large group of small countries in the world than otherwise. In International Organizations the small countries have – as a joint group of countries – a voice in international economic policy. The role of small countries is enhanced if the countries are part of a regional trade integration club.

The bipolar order of the Cold War of 1944–1991 (the year when the Warsaw Pact and the Soviet Union dissolved) was one in which the world market economy was shaped by the United States and its political and military allies. US leadership and the international organizations that had been created in 1944 (IMF/World Bank) and the following decades (e.g. GATT: 1947; WTO: 1995) have helped to achieve the international public good “free trade” and “financial stability”. The OECD as an institution which helped to coordinate fiscal policy among industrialized countries – as did the G7 in an informal way – also played a role.

However, the system failed in preventing the Transatlantic Banking crisis of 2007–09; despite the fact that after the Asian crisis of 1997/98 the IMF had introduced the Financial Sector Assessment Program (FSAP) and despite the warnings of the Bank for International Settlements which since the 1970s has played a crucial role in designing rules for prudential supervision (the Basel Committee on Bank Supervision (BCBS) was created in 1974, the Basel Accord I rules for international banks were adopted in 1988, followed later by Basel II and Basel III). The system thus was augmented by an active G20 – with a first meeting in November 2008 on the Transatlantic Banking crisis - which brought China, India and other countries into a role of more shared international economic policy responsibility.

However, the G20 is a rather heterogeneous group in terms of per capita income of member countries and the size of the countries, involved. The list of promises enshrined in communiqués is long, but there is no clear track that the promises made were held – with the remarkable exception of the G20 Brisbane summit of 2014 when countries promised to increase real GDP by an extra 2% by 2019.

A key question is which alternative international regime would emerge once International Organizations have become rather unimportant, for example by the US bypassing these institutions; and what would be the economic and political consequences of such a development*.* It is most likely that a new grand power regime would be established – with the US, Russia and China being these grand powers and all other countries having to decide which of the grand powers they want to join as a political vassal. The South China Sea will become a major area of new conflict; with the UK and France also sending naval forces to the area although one cannot easily understand what is at stake there for these two European countries. For China, this is a new situation and it might have to accept some European military presence in the area since the key merchandise shipping routes between the EU and Asia will have to be protected by European countries. This will be all the more important as the EU-China and EU-ASEAN trade will grow in the long run, and since NATO is weakening so that Western European countries are less likely to rely on US military protection. A broader EU-China dialogue in all fields of policy would be useful and it is obvious that the EU will push China to remain a supporter of multilateralism. A major problem of China’s international political role is that it has accumulated relatively limited experience in multilateralism over a very short time period, basically starting with WTO membership in 2001, followed by the new Chinese financial policy approach through the AIIB.

## Appendix 9: Maintaining multilateralism: New networking EU-ASEAN-China-X

Multilateralism has been based on International Organizations as they were emerging in a small number in the late 19th century and in much greater number (and role) after 1944. Historical globalization of the 1860s to 1914 created a rapidly growing network of trade and international capital flows and at least in some fields – concerning, for example, postal service, telegraphy and patent protection – European countries plus the US and other countries joined forces to use international law and international organizations to facilitate the exchange of goods, knowledge and information. The leading country of the system was the UK which had lost its global economic leadership around 1900 – with the US becoming the No. 1 in the world economy, but there was no political ambition in the US to play a continued international political role beyond fending off European intervention in Latin America (with the Monroe Doctrine stating the willingness of the US not to accept European interference). The Democratic President Woodrow Wilson, who led the US into World War I and pushed for a new international order in which the League of Nations should play a major role, did not manage to convince the US Senate to follow his plan for this new order so that the US stayed outside. The UK was too weak to provide strong international leadership and Germany as well as Japan left the League of Nations in the 1930s. It was only in 1944 that the US adopted a new strategy and pushed for a multilateral order based on increasingly powerful International Organizations – a strategy that included support for EU integration; a strategy that ended in 2017 with Trump taking office. One may notice that part of the Republican Party stands for a rather isolationist international position and certainly for a deep mistrust in the role of International Organizations which are considered as being dominated by non-US countries and corrupt foreign governments that do not care much about efficient leadership and governance in international organizations.

For the majority of small countries in Asia, Europe, Latin America and Africa, the system of International Organizations and multilateralism is very valuable; as it was also for the US over decades. China seems to support multilateralism and the creation of the Asian Infrastructure Investment Bank– with its seat in Beijing – has been a key step towards a more multilateral (and also regional) approach in China. The US under Obama had adopted TPP in order to create an American-Asian regional integration network that should also establish a counter-balance to China, but Trump had quite different views; or he simply did not understand the logic of TPP and of TTIP. One should note that TTIP – and in a less pronounced way TPP – stand for deep integration approaches that generate benefits in the form of higher trade, higher FDI flows and an acceleration of innovation dynamics in the countries concerned (see on TTIP: Jungmittag and Welfens 2016; the authors use a knowledge production function to analyze the key points of TTIP – and beyond this use a macro production function).

With President Trump, from the non-tradables sector, weakening multilateralism in many ways the question has to be raised: Wither multilateralism? The EU and China as well as ASEAN are three supporters of multilateralism. For China a serious leadership role in this context is quite unusual and it is not clear how strong China’s role could be here. The EU27 will be weakened after BREXIT but it could join political and economic forces with the other regional single market in the world economy, namely ASEAN and push for a very broad cooperation and joint transregional liberalization, for example in the Mercosur-EU-ASEAN triangle. If one would include China in a reliable way, one could imagine that the trilateral regional integration club plus China could be strong enough to fend off Trump’s bilateralism and his determination to bury International Organizations (not only is the WTO on his list for phasing-out, but the Trump Administration has also been very hesitant in 2017 to support the BIS, not to mention leaving the Paris Climate Convention in 2017 and leaving the UN Humanitarian Rights Convention in 2018).

The Asian free trade agreements of the EU are rather weak so far. There is a free trade agreement with Singapore (Kutlina-Dimitrova and Lakatos 2016) and with Vietnam as well as with Japan. However, the talks with other ASEAN countries have been rather slow. A treaty has been signed with Japan in December 2017. Moreover, the EU has a free trade agreement with Korea. EU trade liberalization with China could be possible – not least if one takes into account that small Switzerland has concluded a free trade agreement with China. However, in the case of China the EU should not just consider trade liberalization but investor protection as well. The EU has unique new competences for investment protection treaties since the Treaty of Lisbon (this assignment of competences is based on rather opaque political negotiation dynamics; Meunier 2017).

If one considers a period post-Trump, one could try to envisage a truly trilateral relationship EU-ASIA-USA, namely a new setting which is largely characterized by interdependence (in a nutshell Asia means ASEAN+China+Japan+India). It is unclear which of the existing International Organizations could organize this interdependency in a solid and efficient way.

It is obvious that the perspective of such a triangular interdependency would not make much sense if the EU27 would be unstable so that the final question puts the focus on the perspectives of EU reforms.

## Appendix 10: US merchandise bilateral trade balance (BEA data)

**Table 9:** US Merchandise Bilateral Trade Balance

**Balance on goods**		
**[Millions of dollars]**		
**Source: Bureau of Economic Analysis**		
**Release Date: June 20, 2018**		
**Country**	**2016**	**2017**
Asia and Pacific	−533,849	−558,286
China	−347,270	−375,913
Europe	−166,665	−174,566
European Union	−148,148	−152,597
Euro area	−127,544	−133,953
Other Asia and Pacific	−100,668	−108,204
Mexico	−69,932	−76,127
Japan	−70,378	−69,954
Germany	−65,176	−64,057
Other Euro area	−57,078	−61,495
South and Central America	−52,556	−54,217
Latin America and Other Western Hemisphere	−40,948	−40,974
Italy	−28,713	−31,653
India	−24,440	−22,973
Canada	−16,366	−22,660
Korea, Republic of	−27,433	−22,579
Europe excluding European Union	−18,517	−21,969
Other European Union	−21,150	−21,524
Taiwan	−12,694	−16,192
France	−15,747	−15,527
Members of OPEC	−7473	−13,449
Africa	−4557	−11,547
Other Africa	−2384	−8819
Venezuela	−5771	−8301
South Africa	−2173	−2727
International organizations and unallocated	300	200
Middle East	11,034	339
Luxembourg	1031	659
United Kingdom	546	2879
Argentina	3831	4776
Brazil	5392	9256
Singapore	8679	10,148
Other Western Hemisphere	11,607	13,243
Australia	12,595	14,445
Belgium	14,929	14,776
Other South and Central America	13,924	16,178
Netherlands	23,210	23,344
Hong Kong	27,760	32,936
**Total**	**−1,832,792**	**−1,947,084**

Source: BEA

## Appendix 11: Exposure of leading exporters (selected countries) to the US market

**Table 10:** Exports of Goods and Services from the US in 2017

**Top ten Partner countries**	**Exports of goods and services from the US in 2017, Millions of USD**	**The ratio to US GDP**
Canada	341,309	1.76%
Mexico	276,701	1.43%
China	188,004	0.97%
United Kingdom	126,192	0.65%
Japan	114,746	0.59%
Germany	86,585	0.45%
Korea, Rep.	73,424	0.38%
Brazil	63,489	0.33%
Netherlands	58,744	0.30%
France	52,980	0.27%

Source: BEA

**Table 11:** Exports of Goods and Services to the US (2016)

Countries	Exports of goods and services to the US in 2016, Millions of USD	The ratio to selected countries’ GDP
Mexico	302,863	28.12%
Canada	341,750	22.25%
Korea, Rep.	81,367	5.75%
United Kingdom	131,980	4.98%
Netherlands	37,097	4.77%
Germany	163,669	4.71%
China	416,974	3.73%
Japan	174,891	3.53%
France	63,358	2.57%
Brazil	23,300	1.30%

Note: The data for the exports of services from Mexico and Brazil is not available

Source: UNCTAD, World BankThe ratio of top ten US export destinations’ exports of goods and services to US GDP in the graph above has shown that Canada, Mexico, China, UK, Japan are the five leading US export countries in the sense that these countries depend strongly on the US; Japan and the Republic of Korea, Brazil, the Netherlands and France are also important exporters to the US, but the role of these countries is more modest than the group of the first five countries. In the year before Donald Trump came to power both Mexico and the Canada recorded high relative exports to the US: for these two countries exports exceeded 20% of the respective countries’ own GDP (see figure below). The Republic of Korea, the UK, the Netherlands, Germany and China plus Japan recorded exports that were about 5% of the respective countries’ GDP in 2016 so that Mexico and Canada critically depend on the US. Part of the countries’ links to the US does not go through exports, rather through production of subsidiaries in the United States; in this respect the UK, Germany, the Netherlands, Japan and France have a relatively strong position in the US

## Appendix 12: Economic Growth and Inflation in ASEAN Countries, 2008–2017

As regards economic growth in ASEAN countries there has been some economic convergence over time – except for Brunei Darussalam (major oil exporter) while inflation rates after 2011 have reduced over time and seemed to be anchored around 3% in 2016/2017.

## Appendix 14: Data on UK and US Tariffs

**Table 13:** Import Tariff List of the UK* – For Selected Commodities

**Selected “Most-Favoured Nation” Tariffs as per the UK Govt. in the event of No Deal BREXIT**	**Tariffs; Ad valorum, specific and compound tariffs**
Motor cars and other motor vehicles principally designed for the transport of <10 persons, including station wagons and racing cars, with diesel engine/ with both spark-ignition internal combustion reciprocating piston engine and electric motor as motors for propulsion capable of being charged by plugging to external source of electric power/ with only electric motor for propulsion	10.0%
Motorcycles, including mopeds, with reciprocating internal combustion piston engine of a cylinder capacity <= 250 cm^3^	8.0%
Bananas, fresh (excluding plantains)	114 euros /1000 kg
Bananas, dried (excluding plantains)	16.0%
Fresh or chilled bovine meat, boneless	6.8 + 160.1 euros/100 kg
Natural butter of a fat content, by weight, of > = 80% but <= 85%, in immediate packings of a net content of <= 1 kg (excluding dehydrated butter and ghee)	60.5 euros /100 kg
Grated or powdered cheese, of all kinds	24.9 euros /100 kg
Processed cheese, not grated or powdered, in the manufacture of which no cheeses other than emmentaler, gruyère and appenzell have been used and which may contain, as an addition, glarus herb cheese ‘known as schabziger’; put up for retail sale, of a fat content by weight in the dry matter of <= 56%.	19.1 euros /100 kg
Vanilla, neither crushed nor ground	6.0%
Cloves, whole fruit, cloves and stems, neither crushed nor ground	8.0%
Cocoa paste (excluding defatted)	9.6%
Cocoa butter, fat and oil	7.7%
Fresh or chilled beans ‘vigna spp., phaseolus spp.’, shelled or unshelled	10.4% + 1.6 euros/100 kg
Fresh or chilled boneless cuts of fowls of the species *Gallus domesticus*	61.8 euros /100 kg
Semi-milled round grain rice, parboiled	145 euros /1000 kg
Frozen meat of lambs, boneless, frozen	12.8% + 234.5 euros /100 kg
Fresh or chilled loins and cuts thereof of domestic swine	11.4 euros /100 kg
Pneumatic tyres, new, of rubber, of a kind used for buses or lorries, with a load index of >121	4.5%
Men’s or boys’ swimwear (excluding knitted or crocheted)	12.0%

Note that this UK government guidance on non-preferential tariff rates and quotas on imports if the UK leaves the EU with no deal was officially withdrawn as of 30 January 2020 per the website: https://www.gov.uk/government/publications/temporary-rates-of-customs-duty-on-imports-after-eu-exit

**Table 14:** US Import Tariff Rates on Chinese Goods

Commodity (Top 10 US Imports from China by Value (US$) in 2018	Tariff Rate (as of 15 December 2019)
RADIO TELEPHONES DESIGNED FOR THE PUBLIC CELLULAR RADIOTELECOMMUNICATION SERVICE, EXCLUDING FOR MOTOR VEHICLES	15%
PORTABLE DIGTL AUTOMATIC DATA PROCESSING MACHINES, WEIGHT NOT MORE THAN 10 KG, CONSISTING OF AT LEAST A CENTRAL PROCESSING UNIT, KEYBOARD & A DISPLAY	15%
MACHINES FOR THE RECEPTION, CONVERSION & TRANSMISSION OR REGENERATION OF VOICE, IMAGES OR OTHER DATA, INCLUDING SWITCHING & ROUTING APPARATUS, NESOI	30%
TOYS, PARTS AND ACCESSORIES SUBJECT TO 15 U.S.C. 2052, LABELED OR DETERMINED BY IMPORTER AS INTENDED FOR USE BY PERSONS 3 TO 12 YEARS OF AGE, NESOI	15%
PARTS AND ACCESSORIES OF AUTOMATIC DATA PROCESSING MACHINES AND UNITS NOT INCORPORATING CATHODE RAY TUBE, PRINTING CIRCUIT ASSEMBLIES, MEMORY MODULES	30%
DIGITAL PROCESSING UNITS EXCLUDE SUBHEADING 8471.41 OR 8471.49, MAY CONTAIN IN SAME HOUSING 1 OR 2 OF FOLLOWING: STORAGE, INPUT OR OUTPUT UNITS, NESOI	30%
VIDEO GAME CONSOLES AND MACHINES, OTHER THAN THOSE OF SUBHEADING 9504.30 (COIN OPERATED OR BY OTHER MEANS OF PAYMENT)	15%
MACHINES FOR THE RECEPTION, CONVERSION AND TRANSMISSION OR REGENERATION OF VOICE, IMAGES OR OTHER DATA, NESOI	15%
MONITORS, OF A KIND SOLELY OR PRINCIPALLY USED IN AN AUTOMATIC DATA PROCESSING SYSTEMS OF HEADING 8471, NESOI	15%
PARTS AND ACCESSORIES OF AUTOMATIC DATA PROCESSING MACHINES AND UNITS NOT INCORPORATING A CATHODE RAY TUBE, PRINTED CIRCUIT ASSEMBLIES; NESOI	30%

Source: Own representation of data from Bown (2019), US-China Trade War: The Guns of August, PIIE Trade and Investment Policy Watch blog, Peterson Institute for International Economics (August 26)
